# Effect of soybean protein isolate-*Aronia melanocarpa* extract interaction on the bioaccessibility of anthocyanins after low-pressure cold plasma treatment

**DOI:** 10.1016/j.fochx.2026.104000

**Published:** 2026-05-18

**Authors:** Büşra Yusufoğlu, Rukiye Gundogan, Yeşim Ayık, Oliver Görlitzer, Vildan Enisoğlu Atalay, Esra Capanoglu, Tuba Esatbeyoglu

**Affiliations:** a Department of Chemistry, Faculty of Science and Letters, Istanbul Technical University, Ayazağa Campus, 34469 Istanbul, Türkiye; b Department of Molecular Food Chemistry and Food Development, Institute of Food and One Health, Gottfried Wilhelm Leibniz University Hannover, Am Kleinen Felde 30, 30167 Hannover, Germany; c Department of Food Engineering, Faculty of Chemical and Metallurgical Engineering, Istanbul Technical University, 34469 Maslak, Istanbul, Türkiye; d Department of Bioenginering, Faculty of Engineering and Natural Sciences, Uskudar University, 34662 Usküdar, Istanbul, Türkiye; e Computational Science and Engineering, Istanbul Technical University, İTÜ Ayazağa Campus, Rectorate Building, 34467 Maslak, Istanbul, Türkiye

**Keywords:** Cold plasma, Soybean protein isolate, *Aronia melanocarpa*, Protein–phenolic interaction, Molecular docking

## Abstract

This study evaluated the impact of cold plasma (CP) treatment on the structural and digestive properties of soybean protein isolate (SPI)–*Aronia melanocarpa* extract (AME) complexes. CP treatment significantly modified the secondary structure of SPI–AME complexes, while AME addition enhanced protein stability by increasing α-helix content. FTIR and UHPLC-PDA analyses suggested pronounced structural changes and substantial phenolic changes, particularly for chlorogenic acids and cyanidin derivatives, throughout the digestive process. Anthocyanin levels increased in the gastric phase due to flavylium cation stabilization but declined significantly at intestinal pH 7.0. CP-treated complexes showed 84-89% anthocyanin retention compared to free AME, which showed a decline to 2-3%. Molecular docking further supported these findings, revealing strong binding affinities between cyanidin derivatives and glycinin (1FXZ), explaining the enhanced complex stability. These results indicate that CP-mediated protein-polyphenol interactions successfully protect sensitive bioactives during digestion, offering a promising approach for developing stable functional food systems.

## Highlights

• SPI-*Aronia* extract interaction presents a promising system for the development of functional foods.
• Cold plasma (CP) application alters the structure of protein–anthocyanin interactions and improves digestibility.
• Increased protein concentration significantly alters the stability of anthocyanin–protein complexes.

## Graphical abstract

This figure was prepared using Freepik, Powerpoint and ChemSketch applications.

**Figure:**
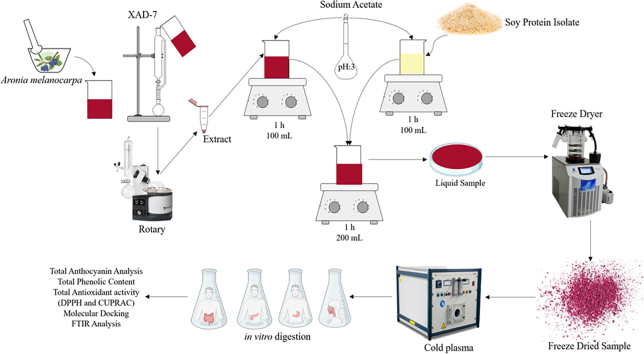

## 1 Introduction

Soybean protein is an important plant protein source widely used in the food industry because of its functional properties and nutritional value. Its commercial form is soybean protein isolate (SPI), is composed mainly of β-conglycinin (7S) and glycinin (11S) and contains approximately 90% protein (Wang, Pham, & Adhikari, 2024; Zhang et al., 2018). SPI is non-toxic, has low allergenic potential, has excellent techno-functional properties, such as gel formation, emulsification ability, and water- and oil-retention capacity, and is considered an excellent carrier for different bioactive compounds (Akharume et al., 2021; Dumitrascu et al., 2020). Owing to these features, SPI is a physiologically beneficial product, recognized by the Food and Drug Administration (FDA) for its role in reducing cholesterol levels and the risk of coronary heart disease (CHD) under the Nutrition Labelling and Education Act of 1990 (Dumitrascu et al., 2020; Shubhashini et al., 2022).

Recently, cold plasma (CP) has become one of the most popular non-thermal food processing techniques in the food industry. It contains various active species that are formed as a result of the ionization of a gas (Mravlje et al., 2021; Oner et al., 2023). CP technology is integrated into various stages of food production, from processing to packaging. Its primary applications include enhancing the stability of bioactive compounds, reducing reactive oxygen species (ROS), reactive nitrogen species (RNS), and carbonyl species (RCS), and preserving microbial threats such as mycotoxins, spores, viruses, and pesticides to extend shelf-life (Misra et al., 2014).

CP application modifies protein structure through oxidative mechanisms, inducing partial unfolding and molecular flexibility. This promotes protein fibrillation by exposing reactive sites and increasing the availability of amino groups for stronger interactions (Song et al., 2026). These fibrillar proteins can further interact with polysaccharides to form stable complexes with improved interfacial properties. In addition, CP enhances protein reactivity and increases the availability of amino groups and may facilitate stronger interactions with polysaccharide carbonyl groups. This enables CP-assisted glycation to proceed more efficiently than conventional thermal methods, leading to faster reaction kinetics and improved formation of functional protein–polysaccharide conjugates (Ge et al., 2026). Moreover, CP-assisted acid–heat treatment has been shown to induce the formation of β-lactoglobulin fibrils that self-assemble with fucoidan into structured complexes. These systems create thick and compact interfacial layers around oil droplets in emulsions, thereby enhancing physical stability. Consequently, bioactive compounds such as lycopene exhibit improved protection against environmental stressors, higher retention, and increased bioavailability, while the dense network structure also contributes to controlled release behavior (Song et al., 2025).

Black chokeberry (*Aronia melanocarpa* L.) is frequently recognized as a ‘superfood’ due to its richness in phenolic compounds, such as proanthocyanidins, anthocyanins, hydroxycinnamic acids, flavonol glycosides, and quercetin glycosides. *Aronia melanocarpa* is considered one of the richest known sources of anthocyanins (Cho et al., 2023; Öztürk, 2025). The major anthocyanins reported in *Aronia melanocarpa* are cyanidin 3-*O-*glucoside, cyanidin 3-*O-*xyloside, cyanidin 3-*O*-arabinoside, and cyanidin 3-*O*-galactoside (Xie et al., 2024). Beyond its rich profile of proanthocyanidins and flavonol glycosides, AME exhibits potent antioxidant, anti-inflammatory, and antiproliferative activities (Gao et al., 2021; Ngum et al., 2023). These health-promoting properties contribute to its effectiveness against oxidative stress and its potential cytotoxic effects on cancer cells, making it a valuable functional food ingredient (Cho et al., 2023; Zhang et al., 2018).

The main objective of this study was to determine how CP treatment influences SPI-AME interactions under in vitro digestion conditions. The SPI–AME combination offers a suitable model system for evaluating enhanced phenolic stability during digestion and improved potential bioavailability through protein–polyphenol complexation. In this context, AME was first enriched with polyphenols using XAD-7 resin, thus providing a clearer understanding of the role of low molecular weight phenolic compounds in protein-phenolic interactions. The XAD-7 column was used in this study not as a process or application alternative, but for the selective purification of the phenolic fraction and a more accurate, targeted evaluation of interaction mechanisms. CP treatment was applied to investigate how surface and chemical modifications in proteins and phenolic compounds influence SPI–AME complex formation, digestion stability, and potential bioavailability without causing thermal degradation. In this respect, the study aims to comprehensively evaluate the regulatory role of CP technology on plant protein-phenolic systems and the behavior of these interactions in the digestive process.

Despite extensive studies on protein-polyphenol interactions, the application of CP to regulate these interactions remains an insufficiently explored area. While conventional modification methods often cause thermal degradation of sensitive anthocyanins, there is a critical knowledge gap regarding how CP-induced structural unfolding and chemical modifications affect the binding affinity and digestive bioavailability of *Aronia melanocarpa* anthocyanins. Understanding these mechanisms is crucial for the development of next-generation functional foods with enhanced stability. In this context, the present study focuses on the effects of cold plasma treatment on the structural properties, digestion behavior, and anthocyanin bioavailability of SPI-AME systems. The findings are interpreted as evidence of structural-functional modifications and potential interaction trends, rather than definitive proof of covalent interaction mechanisms. Therefore, this study aims to address this gap by systematically evaluating the impact of CP treatment on SPI–AME complexes, focusing on structural transformations, interaction mechanisms, and bioaccessibility under in vitro digestion conditions. To ensure a targeted evaluation of these mechanisms, AME was first enriched using XAD-7 resin for the selective purification of the phenolic fraction. Overall, this research provides a comprehensive insight into the role of CP technology in enhancing the stability and potential bioavailability of plant protein-phenolic systems and presents a model for the development of next-generation functional foods.

## 2 Materials and methods

### 2.1 Materials

*A. melanocarpa* pomace was kindly provided by Aronia Original (Dresden, Germany). *Aronia* 20% (A20), a commercial *A. melanocarpa* juice extract containing 20% anthocyanins, was obtained from Symrise (Holzminden, Germany). SPI was purchased from Taste Market (Hannover, Germany).

### 2.2 Chemicals

Ultrapure water was obtained by PURELAB® flex 3 (ELGA LabWater, Veolia Water Technologies Deutschland, Celle, Germany). Amberlite XAD-7, ABTS (2,2′-azino-bis-(3-ethylbenzothiazoline-6-sulfonic acid)), DPPH (2,2-diphenyl-1-picrylhydrazyl), ethanol (HPLC grade), sulforhodamine B and trypsin were purchased from Sigma-Aldrich (Steinheim, Germany). Acetonitrile (HPLC grade) was purchased from Honeywell (Seelze, Germany). Acetic acid (HPLC grade) was purchased from VWR International (Darmstadt, Germany). Folin-Ciocalteu reagent, dimethyl sulfoxide (p.a.) and casein soy peptone broth (CASO broth) were purchased from Merck (Darmstadt, Germany). HPLC-grade methanol was obtained from Chemsolute (Th. Geyer, Renningen, Germany). Chlorogenic acid (≥ 95%) was procured from Carl Roth (Karlsruhe, Germany), whereas cyanidin-3-*O*-galactoside chloride (≥ 95%) was obtained from PhytoLab (Vestenbergsgreuth, Germany). Ethanol (denatured with methyl ethyl ketone, 99%) was purchased from Walter-CMP (Kiel, Germany). Potassium peroxodisulfate (K_2_S_2_O_8_) was obtained from Riedel-de-Haën (Seelze, Germany). Gallic acid monohydrate and 6-hydroxy-2,5,7,8-tetramethylchroman-2-carboxylic acid (Trolox, ≥98% purity) were purchased from Fluka (Buchs, Switzerland). Formic acid (HPLC grade), sodium carbonate (≥99%, p.a.), sodium chloride (≥99%, p.a.), sodium hydroxide (≥99%, p.a.), trichloroacetic acid, tris(hydroxymethyl)aminomethane and casein soy peptone agar (CASO agar) were purchased from Carl Roth (Karlsruhe, Germany).

### 2.3 Preparing *Aronia melanocarpa* L. extract via XAD-7 column chromatography

**Fig. 1:**
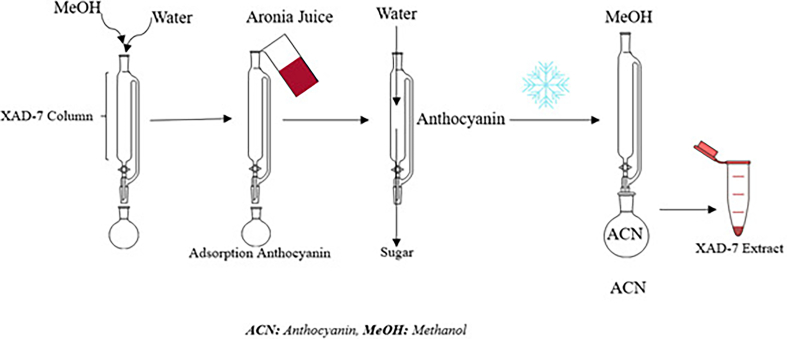
Schematic view of XAD-7 column chromatography.

The enrichment of polyphenols from *Aronia* juice was performed using Amberlite® XAD-7 (100 cm × 6 cm) column according to a modified method of Esatbeyoglu et al. (2017). The XAD-7 column was initially conditioned with 0.5 L of methanol, followed by equilibration with 1 L deionized water. Freshly prepared *Aronia* juice, filtered through cheesecloth, was loaded onto the column at a controlled flow rate. Amberlite XAD-7 is a non-ionic adsorbent resin that enables the retention of phenolic compounds, including anthocyanins, through hydrophobic interactions. To remove polar non-phenolic compounds such as sugars, organic acids, and salts, the column was washed with deionized water. Subsequently, the adsorbed phenolic fraction, primarily consisting of anthocyanins, was eluted using a mixture of methanol/acetic acid (19:1, *v/v*). The collected eluate was concentrated under vacuum using a rotary evaporator and subsequently freeze-dried to obtain a stable powder extract (AME). The purified AME was stored for subsequent analysis. A schematic representation of the purification procedure is presented in Fig. 1.

### 2.4 Preparing interaction solutions

The SPI and AME solutions were prepared in sodium acetate buffer (1 M) at pH 3.0. These solutions were combined at volumetric ratios of 1:1, 2:1, and 4:1 *(v/v)* and stirred at 500 rpm for 1 h at room temperature (25 °C). AME solutions were prepared by diluting the extract to a final concentration of 0.3% (*w/v*) with sodium acetate buffer (1 M). The mixtures were blended by a magnetic stirrer for 2 h to ensure complete dispersion. The samples were subsequently transferred into Petri dishes and subjected to freeze-drying. The lyophilized samples were then stored at −20 °C until further analysis.

### 2.5 Preparation of methanolic extracts

The extraction of samples for total phenolic content, antioxidant activity and individual phenolic compound analyses was performed according to the methods described by Capanoglu et al. (2008). 5 mL of 75% methanol with 0.1% formic acid were added to each 1 g sample and sonicated for 15 min. The supernatant was collected after centrifugation (NuAire, Hitachi, US) at 2500 rpm for 10 min. Another 5 mL of 75% methanol was added to the residue, and the extraction step was repeated. Both supernatants were collected in another test tube and adjusted to a final volume of 10 mL (Capanoglu et al., 2008).

### 2.6 Cold plasma treatment

The low-pressure CP system operated under reduced pressure conditions using ambient air as the processing gas and a capacitive discharge mechanism consisting of eight electrodes arranged in a circular chamber. Each electrode measured 100 mm in length. The system incorporated a rotary chamber operating at 10 rpm, while the plasma was generated at a frequency of 13.56 MHz and a maximum power output of 100 W. The plasma chamber was constructed from borosilicate glass. Inside the chamber, four rotary columns enabled continuous rotation of the sample vials (Femto type; Diener Electronic, Ebhausen, Germany). A rotary pump with an intake capacity of 6 m³/h was used to evacuate the chamber (Pfeiffer, Asslar, Germany).

SPI, AME, and SPI-AME mixtures (1:1, 2:1, and 4:1 *(v/v)*) in powder form were weighed and transferred into 200 mL square-shaped bottles, with 5 g of each sample placed in each bottle. Plasma treatment was performed at room temperature under rotational conditions, with a power setting of 80 W and a pressure of 0.60 mbar for 10 min. The selected CP parameters were chosen to provide sufficient energy for surface modification of SPI while minimizing potential thermal and oxidative degradation of anthocyanins in AME (Amorim et al., 2023; Laroque et al., 2022; Rout & Srivastav, 2024).

### 2.7 Zeta potential (net surface charge) measurement

The net surface charge (zeta potential) for each sample was determined by the method of Gundogan and Karaca (2020) as a function of pH 3.0 using a Nano-ZS (Malvern Instruments, Worcestershire, UK) based on the electrophoretic motion of protein solutions. For this purpose, a 0.05% *(w/v)* sample solution was prepared with pure water, and the pH was adjusted with 1.0 M NaOH and 1.0 M HCl.

### 2.8 Color analysis

To evaluate color changes in CP-treated and untreated samples, a CM-5 spectrophotometer (Konica Minolta, Langenhagen, Germany) was used. The CIE color parameters were expressed as L*, a*, b*. Here, L* represents lightness of the samples: 100 is white, and 0 is black. The parameter a* represents the color distribution between green (negative a*) and red (positive a*). Similarly, negative values of b* represent blue color, and positive values indicate yellow color.

### 2.9 Fourier transform infrared spectroscopy (FTIR)

FTIR measurements of SPI and SPI–anthocyanin complexes were performed using a Tensor II FTIR spectrophotometer (Bruker Scientific LLC, Billerica, USA) according to the methods of He et al. (2018). Measurements of the powdered samples were carried out over the wavenumber range of 400–4000 cm^−1^. Band components were identified based on their characteristic average wavenumbers.

Quantitative analysis of the protein secondary structure was performed using a hybrid approach combining OriginPro 2021 (OriginLab Corporation, Northampton, MA, USA) for initial spectral conversion, baseline correction, smoothing via a Savitzky–Golay filter and visualization, with custom Python (v3.x) scripts for data processing and deconvolution. Absorbance (A) data were processed in Python using the NumPy and SciPy libraries (Python Software Foundation), and the Amide *I* region (1700–1600 cm^−1^) was precisely identified using second-derivative spectroscopy. The Amide *I* band was then deconvoluted by fitting multiple Gaussian functions based on the detected centres. Finally, the relative proportions of secondary structures (α-helix, β-sheet, turns, and random coil) were determined by normalizing the area under each fitted component peak to the total area of the Amide *I* band.

### 2.10 Spectrophotometric assays

The total phenolic content (TPC) was determined according to the method described by Singleton and Rossi (1965). The results were expressed as mg of gallic acid equivalents (GAE) per 100 g of dry weight.

The total antioxidant capacity was determined via two different assays. Copper (II)-reducing antioxidant capacity (CUPRAC) and DPPH assays. The results were expressed as mg of Trolox equivalents (TE) per 100 g of dry weight (Apak et al., 2004; Kumaran & Joel karunakaran, 2006).

Total anthocyanin content (TAC) measurement was carried out according to the method of Giusti and Wrolstad (2001), in which buffer solutions of 0.025M KCl at pH 1.0 and 0.4 M sodium acetate at pH 4.5 were prepared, and quantitative analysis was performed on the basis of the difference in absorbance (Giusti & Wrolstad, 2001).

### 2.11 *In vitro* digestion procedure

A standardized static *in vitro* digestion method was applied (Brodkorb et al., 2019). The samples were passed through a gastrointestinal tract model that simulated the mouth, stomach and small intestinal digestion phases. For the oral phase, samples were homogenized with simulated saliva; human salivary α-amylase (1500 U/mL) solution in simulated salivary fluid (SSF), calcium chloride (CaCl_2_), and H_2_O were added (all reagents were pre-incubated at 37 °C). After being adjusted to pH 7.0, the mixture was agitated and incubated for 2 min at 37 °C. For the gastric phase, the oral mixture was mixed with the simulated gastric fluid, porcine pepsin solution (25,000 U/mL), and CaCl_2_, and the pH was adjusted to 3.0 with 1 M HCl. The mixture was incubated at 37 °C in a shaker at 100 rpm for 2 h. For the intestinal phase, the gastric fluid was mixed with the simulated intestinal fluid, pancreatin (800 U/mL), and bile salts (160 mM), and the pH was adjusted to 7.0 with 1 M NaOH. The mixture was incubated at 37 °C in a shaker at 100 rpm for 2 h. After the intestinal phase, the enzymatic reaction was immediately stopped by cooling the samples in an ice bath for 10 min. Subsequently, the mixtures were centrifuged at 23,000×*g* for 10 min at 4 °C, and the supernatants were stored at −20 °C until further analysis. The bioaccessibility of individual phenolics in samples is used to determine their release and absorption in the gastrointestinal tract. The percent bioaccessibility of compounds in the intestinal and gastric phases of *in vitro* digestion was calculated by comparing the amount of compound in the undigested sample with the amount in the digested sample, and this calculation was performed from the peak areas determined at λ = 280, 320 and 520 nm according to the method described in a study by Ozkan et al. (2025).

### 2.12 Quantitative determination of phenolic compounds via UHPLC-PDA analysis

The analysis of the samples was conducted with modifications based on Rodríguez-Werner et al. (2019), using an Agilent 1290 UHPLC system (Waldbronn, Germany) equipped with a G7117B photodiode array (PDA) detector, a G7120A high-speed pump, a G1316A column oven, and a G7167B autosampler. The separation was performed on a Luna Omega Polar C18 column (1.6 μm, 100 Å, 150 × 2.1 mm) with a guard column made of the same material (Phenomenex, Aschaffenburg, Germany). The mobile phase consisted of 2% formic acid in water (solvent A) and 2% formic acid in acetonitrile (solvent B). The following gradient with a flow rate of 0.3 mL/min was applied 5–25% B (0.0 to 16.0 min) and 25–95% B (16.0 to 19.1 min), followed by 95% B held until 21.0 min. After this, a re-equilibration period of 4 min was conducted. Detection was performed at λ = 280, 320, and 520 nm, with a column temperature of 30 °C. The quantification of phenolic acids was conducted as chlorogenic acid equivalents at λ = 320 nm, utilizing an external calibration (range: 0.77–245.75 mg/L; R^2^ > 0.999; LOD: 0.22 mg/L; LOQ: 0.96 mg/L). The presence of anthocyanins was detected at a wavelength of λ = 520 nm and subsequently quantified as cyanidin-3-*O*-galactoside equivalents (range: 0.72–231.33 mg/L; R^2^ > 0.999; LOD: 0.28 mg/L; LOQ: 1.24 mg/L). Peak identification was performed according to the retention times and spectra, as described by Rodríguez-Werner et al. (2019). All UHPLC data were measured in technical duplicates.

### 2.13 *In silico* evaluation of interactions between soy protein and *Aronia melanocarpa* extract

Molecular docking is a method that is frequently used to predict the most preferred orientation of a ligand within a protein binding site to form a stable protein–ligand complex. Furthermore, it is a good tool with scoring functions to anticipate the binding affinities of ligands or the strength of connections between proteins and ligands (Rauf et al., 2015). This study also aimed to analyze the interactions of cyanidin and several anthocyanins (anthocyanidin-3-*O*-glycosides) with SPI. Accordingly, docking simulations were performed as described below.

3D optimized structures of the ligands were prepared via the SPARTAN’14 program (Hehre, 2003, Wavefunction, Inc, 2014). To obtain the structures, conformer distributions were obtained via the semiempirical PM6 method (Stewart, 2007; Stewart, 2008), and all rotatable bonds were rotated 6 times (at 60° angles). Subsequently, geometry optimization was performed via the ab initio HF/6-31+G (d,p) method (Roothaan, 1951; Valatin, 1961) on the Gauss View 5.0 (Dennington et al., 2009) and Gaussian’09 software packages (Frisch et al., 2010). The optimized ligands were prepared in AutoDockTools 1.5.6 using the Gasteiger charge calculation method (Sanner, 1999) and saved as pdbqt files for docking studies. The crystal structure of the SPI (glycinin, PDB ID: 1FXZ) was acquired from the RCSB Protein Data Bank (Berman et al., 2000). The structure was also prepared by setting a 40 x 40 x 40 Å^3^ grid box for docking studies on AutoDockTools 1.5.6 as a PDBQT file. Finally, the binding energy of each ligand to 1FXZ was obtained via AutoDock Vina (Trott & Olson, 2010) via 3 trials, and the molecular interactions with the amino acids in the active site were visualized via Discovery Studio Visualizer (Dassault Systèmes BIOVIA, 2017).

### 2.14 Statistical analysis

All measurements were performed in triplicate. One-way analysis of variance (ANOVA) with a Scheffé post hoc test was used to measure significant differences in physicochemical and functional properties as a function of treatment groups. All the statistical analyses were performed via statistical software (SPSS Statistics 25.0 for Windows version, IBM, New York, United States). Statistical significance was accepted at *p* < 0.05.

## 3 Results and discussion

### 3.1 Zeta potential (net surface charge) measurement

**Fig. 2:**
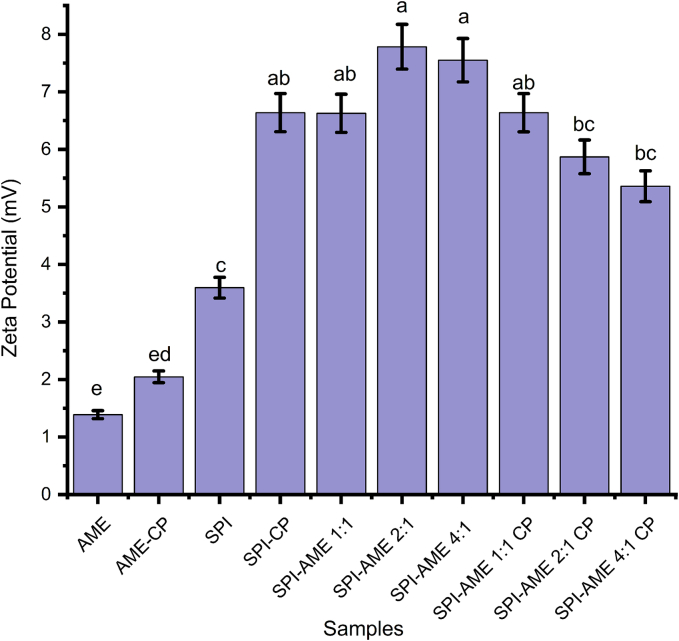
Surface charge of CP-treated vs. untreated soy protein–anthocyanin complexes. Data represent the mean ± SD (*n* = 3). Different lowercase letters in the different bars indicate significant differences (*p* < 0.05). AME: *Aronia melanocarpa* extract, SPI: soybean protein isolate, CP: cold plasma, AME-CP: cold plasma-treated AME, SPI-CP: cold plasma-treated SPI, SPI-AME 1:1: SPI-AME interaction at a 1:1 ratio *(v/v)*, SPI-AME 2:1:SPI-AME interaction at a 2:1 ratio *(v/v)*, SPI-AME 4:1: SPI-AME interaction at a 4:1 ratio, SPI-AME CP 1:1: cold plasma-treated-SPI-AME interaction at a 1:1 ratio *(v/v)*, SPI-AME CP 2:1: cold plasma-treated-SPI-AME interaction at a 2:1 ratio *(v/v)*, AME CP 4:1: cold plasma-treated-SPI-AME interaction at a 4:1 ratio *(v/v)*.

The structural and colloidal stability of SPI-AME complexes (protein-to-polyphenol ratio of 1:1, 2:1, and 4:1 *(v/v)* formed at pH 3.0 was investigated following CP treatment. Zeta potential (ZP) was measured as a key indicator of surface charge modifications and the resulting colloidal stability of these protein-polyphenol systems. As shown in Fig. 2, ZP analysis indicated that both SPI-AME interaction and CP treatment significantly altered the surface charge of the protein. Untreated AME and SPI exhibited inherent surface charges, which underwent distinct shifts upon complexation at different ratios (SPI-AME ratios of 1:1, 2:1, and 4:1 *(v/v)*).

The SPI-AME 2:1 and 4:1 *(v/v)* complexes exhibited a pronounced changes in zeta potential, suggesting interactions that may enhance colloidal stability. This observation is consistent with Jiang et al. (2019), who reported that the complexation of denatured SPI with anthocyanin increased the absolute value of the zeta potential in comparison with that of soy protein and thus increased the colloidal stability. CP treatment further altered the surface charge (p < 0.05), likely due to structural reorganization and oxidation of surface functional groups that modulate the electrical double layer. These findings are consistent with the literature, which reported that CP treatment is an effective technique for modulating the surface characteristics and stability of food-grade protein complexes. For example, Deng et al. (2025) indicated that atmospheric CP treatment significantly increased the surface charge of walnut gluten under optimal conditions, followed by a decrease after prolonged treatment.

Specifically, reactive oxygen and nitrogen species generated during plasma treatment can interact with amino acid side chains on the protein surface, leading to the formation of additional acidic functional groups through oxidation (Bayati et al., 2026). This oxidative modification initially tends to increase the net negative charge on the protein surface. However, in the presence of AME, the positively charged flavylium cation form of anthocyanins (stable at pH 3.0) interacts with these newly formed or exposed negative sites on the soy protein. This interaction results in a shift toward lower absolute zeta potential values. This interpretation is supported by the molecular docking findings of Zheng et al. (2021). Their study indicated that the amino acid binding pockets of soybean 7S and 11S globulins can encapsulate anthocyanin monomers through electrostatic and hydrogen bonding, thereby altering the exposed surface charge. This mechanism supports the hypothesis that the interaction between the flavylium cations and the SPI surface reduces the net absolute zeta potential. The reduction in net surface charge provides additional evidence supporting the formation of SPI-AME complexes facilitated by CP-induced surface activation (Bayati et al., 2026). Consequently, the further decrease in absolute zeta potential values observed with prolonged treatment in this study may be associated with increased surface oxidation and subsequent phenolic binding, which promotes greater charge stabilization within the complex.

### 3.2 Color analysis

CP treatment significantly influenced the color parameters (L*, a*,b*) of the SPI-AME complexes (*p* < 0.05), as shown in Table 1. SPI presented the highest L^⁎^ values (lightness), indicating a brighter appearance, which significantly increased following CP treatment (SPI-CP) (80.21 to 82.57). This lightening effect may result from plasma-induced surface modifications and structural alterations that enhance light reflection. In addition, AME alone presented substantially lower L* values due to its intense pigmentation. Following CP treatment (AME-CP), the L* values slightly increased, suggesting that plasma reactive species may partially induce pigment degradation or structural shifts that affect light absorption.

**Table 1:** Color parameters (L*, a*, b* values) of cold plasma-treated and untreated soy protein–anthocyanin complexes.

Samples	L*	a*	b*
SPI	80.21 ± 0.08^b^	0.01 ± 0.008^f^	18.88 ± 0.14^b^
SPI-CP	82.57 ± 0.02^a^	0.14 ± 0.02^f^	20.06 ± 0.02^a^
AME	19.37 ± 0.28^h^	10.7 ± 0.24^c^	1.63 ± 0.01^e^
AME-CP	22.33 ± 0.02^g^	11.2 ± 0.07^e^	1.67 ± 0.04^c^
SPI-AME 1:1	36.24 ± 0.54^f^	23.2 ± 0.17^d^	−3.32 ± 0.04^f^
SPI-AME 1:1 CP	37.94 ± 0.07^e^	24.1 ± 0.09^d^	−2.32 ± 0.04^e^
SPI-AME 2:1	35.48 ± 0.53^f^	33.3 ± 0.29^b^	−2.45 ± 0.009^e^
SPI-AME 2:1 CP	37.02 ± 0.02^e^	34.3 ± 0.00^a^	−1.45 ± 0.009^d^
SPI-AME 4:1	42.47 ± 0.37^d^	31.6 ± 0.48^c^	−4.34 ± 0.009^f^
SPI-AME 4:1 CP	43.93 ± 0.04^c^	32.7 ± 0.04^b^	−3.34 ± 0.009^f^

AME: *Aronia melanocarpa* extract, SPI: soybean protein isolate, CP: cold plasma, AME-CP: cold plasma-treated AME, SPI-CP: cold plasma-treated SPI, SPI-AME 1:1: SPI-AME interaction at a 1:1 ratio *(v/v)*, SPI-AME 2:1:SPI-AME interaction at a 2:1 ratio *(v/v)*, SPI-AME 4:1: SPI-AME interaction at a 4:1 ratio *(v/v)*, SPI-AME CP 1:1: cold plasma-treated-SPI-AME interaction at a 1:1 ratio *(v/v)*, SPI-AME CP 2:1: cold plasma-treated-SPI-AME interaction at a 2:1 ratio *(v/v)*, AME CP 4:1: cold plasma-treated-SPI-AME interaction at a 4:1 ratio *(v/v)*. Data represent the mean ± SD (*n* = 3) (*p* < 0.05). Different lowercase letters in the same column indicate significant differences (p < 0.05).

In SPI-AME mixtures, the incorporation of anthocyanins markedly reduced L* values compared to SPI alone, with the SPI-AME 1:1 (*v/v*) formulation exhibiting the most pronounced decrease due to higher anthocyanin content. However, CP treatment consistently mitigated this reduction across all ratios, with the SPI-AME 4:1 (*v/v*) CP complex exhibiting a particularly pronounced increase in lightness. This enhanced lightening effect is potentially linked to structural rearrangements in both the protein and anthocyanin matrices, especially at higher protein concentrations. This observation suggests that plasma-induced molecular interactions between SPI and AME may contribute to color stabilization or alter pigment distribution through structural rearrangements.

The a* (redness) and b* (yellowness) values increased across all formulations after CP treatment, reflecting an intensified color expression. This is suggested to be linked to enhanced anthocyanin stabilization or increased chromophore exposure due to plasma-induced modifications of the protein matrix, facilitating stronger pigment-protein interactions. The highest b* values were observed for the SPI-AME 1:1 CP (*v/v*) complex, suggesting that the combination of high anthocyanin content and plasma treatment influences yellowness, potentially through pigment transformation reactions or changes in pigment accessibility. Unlike traditional treatments, CP stands out for its potential to maintain the stability of natural pigments, such as anthocyanins and carotenoids (Ramezan et al., 2024). Collectively, these findings highlight the potential of CP treatment to modulate the visual and structural properties of protein–anthocyanin complexes (Bayati et al., 2024; Farias et al., 2020).

### 3.3 FTIR spectroscopy

FTIR spectroscopy was used to analyze structural changes associated with protein-phenolic interactions by monitoring molecular vibrations, such as stretching and bending, which generate characteristic absorption spectra (Siddique, 2024). The FTIR spectra of SPI are shown in Fig. S1. The amide I region (approximately 1600–1700 cm^−1^) is highly sensitive to protein secondary structure and is commonly associated with β-sheet, α-helix, random coil, and β-turn conformations (Luo et al., 2022). This band is characteristic of protein secondary structures. Previous research revealed the following secondary structures in the amide I region: α-helix, approximately 1650–1651 cm^−1^; β-sheet, 1615–1640 cm^−1^; random coil, 1641–1649 cm^−1^ and β-turn, 1660–1688 cm^−1^ (Shi et al., 2002). For the amide II band, the SPI presented a peak at approximately 1515.67 ± 2.7 cm^−1^. The amide II band is primarily attributed to N–H bending (40–60%) and C–N stretching (18–40%) vibrations within the wavenumber range of 1480–1585 cm^−1^ (Li et al., 2024). Amide III indicates interactions between proteins and other macromolecules, such as carbohydrates, with C—N stretching and N—H bending vibrations (1239.4 cm^−1^).

Following CP treatment (SPI-CP) (Fig. S1a), some modifications, such as alterations in hydrogen bonding (3280.9–3269.8 cm^−1^) and protein structural reorganization and conformational modification, occurred, as shown in Fig. S1a. The amide I and II peaks slightly shifted and increased in intensity, while the shift in the amide III region (from 1234.4 to 1239.4 cm^−1^) may reflect modifications in the local chemical environment of amide groups following CP treatment. The C—H (2929.1–2926.1 cm^−1^) and C—O (1071.1–1068.8 cm^−1^) bands did not change significantly, indicating that the aliphatic chains remain largely unaffected. The FTIR spectrum of AME showed characteristic anthocyanin peaks, including broad O—H stretching (3200–3500 cm^−1^), conjugated C=O bonds (1650–1750 cm^−1^), aromatic ring vibrations (1500–1600 cm^−1^), and glycosidic C—O bonds (1000–1300 cm^−1^) (Fig. S1a). The strong peak at 1650–1750 cm^−1^ suggests the presence of conjugated carbonyl (C=O) bonds, which are commonly found in anthocyanins. The peak at 1000–1300 cm^−1^ suggested the presence of ether or glycosidic bonds, and after the CP effect, changes in these peaks could indicate alterations in anthocyanin glycosylation.

In the SPI-AME (1:1) complexes, the shift of amide I from 1633.6 cm^−1^ to 1626.8 cm^−1^ and amide III from 1239.4 to 1229.9 cm^−1^ suggests protein-polyphenol interactions, likely involving cross-linking (Fig. S1b). In the case of the SPI–AME CP interaction (1:1) *(v/v)* in Fig. S1c, CP slightly increased the shift in hydrogen bonding and indicated more pronounced shifts in the amide I and II bands, suggesting enhanced protein structural reorganization. Furthermore, a new aromatic peak emerged at 1443.7 cm^−1^ following CP treatment, which was absent in the untreated complexes (Fig. S1c). This observation suggests that CP-induced reactive species facilitate the oxidation or polymerization of AME phenolics, possibly leading to phenolic modifications. The shifts in C—O and C—N stretching (from 1052.8 to 1067.0 cm^−1^) indicate changes in molecular interactions, including hydrogen bonding and other non-covalent associations. As the protein-to-polyphenol ratio increases (e.g., 4:1), the relative decrease in phenolic content leads to weaker hydrogen bonding and more localized modifications; thus, this indicates that a specific stoichiometry is necessary to maximize plasma-derived molecular interactions. While the FTIR spectra provide evidence of structural modifications and molecular interactions, Table 2 further characterizes the effects of CP treatment and protein–anthocyanin interactions on protein binding behavior. In this context, the effects of both CP and protein-anthocyanin interactions were examined.

**Table 2:** Secondary structure distribution (%) of cold plasma-treated vs. untreated soy protein (SPI)–anthocyanin (AME) complexes.

Structure	SPI	SPI-CP	SPI-AME 1:1	SPI-AME 1:1 + CP	SPI-AME 2:1	SPI-AME 2:1 + CP	SPI-AME 4:1	SPI-AME 4:1 + CP
β-Sheets	55.78	53.29	56.83	54.27	57.50	56.1	57.13	55.4
α-Helix	13.88	16.78	14.33	15.92	12.81	12.7	14.31	15.37
Random coil	28.04	27.33	26.83	27.8	27.75	29.1	26.19	27.29
β-Turn	2.28	2.59	1.20	1.99	1.92	2.1	2.25	1.96

SPI: Soybean protein isolate, SPI-CP: Cold plasma treated-SPI, SPI-AME 1:1: SPI-AME interaction at 1:1 *(v/v)*, SPI-AME CP 1:1: Cold plasma treated-SPI-AME interaction at 1:1 ratio *(v/v)*, SPI-AME 2:1: SPI-AME interaction at 2:1 ratio *(v/v)*, SPI-AME CP 2:1: Cold plasma treated-SPI-AME interaction at 2:1 ratio *(v/v)*, SPI-AME 4:1: SPI-AME interaction at 4:1 ratio *(v/v)*, SPI-AME CP 4:1: Cold plasma treated-SPI-AME interaction at 4:1 ratio *(v/v)*.

The deconvolution of the amide I band revealed that AME concentration and CP treatment induced a concentration-dependent conformational modulation of SPI (Table 2 and Fig. S2). The impact of AME concentration on disordered structures followed a non-linear trend. As the AME concentration initially decreases in the SPI-AME mixture, the disordered structures (26.83 → 27.75% for random coil and 1.20 → 2.25% for beta turn) increased. However, a slight reduction in random coil content was observed at the 4:1 ratio (*v/v*) (26.19%), which can be attributed to increased intermolecular interactions and radical-induced cross-linking, leading to a degree of microscopic aggregation (Sharath Kumar et al., 2024). This suggests that anthocyanin concentration influences the conformational state of SPI, with the extent of unfolding depending on the protein-to-anthocyanin ratio.

The decrease in α-helix content and the concomitant increase in random coil content can be attributed to a decrease in the number of intramolecular hydrogen bonds or to the opening of the SPI molecule and subsequent formation of the random coil structure (Yan et al., 2021). Combined evaluation of protein-phenolic interaction with cold plasma treatment further promoted this structural shift, leading to a significant decrease in β-sheet content compared to protein-phenolic interaction alone. This reduction is critical as low amounts of β-sheets indicate a decrease in the number of hydrogen bonds between peptide chains (Hou et al., 2025).

Cold plasma generates reactive oxygen and nitrogen species, such as hydroxyl radicals (•OH), which can modify protein surfaces through oxidation and etching-related processes. These species can induce the formation of protein radicals, leading to the oxidation of amino acid side chains and the disruption or rearrangement of noncovalent interactions. Furthermore, these radicals may promote protein modification reactions, generating additional reactive sites within the protein matrix (Sharath Kumar et al., 2024). These reactive components weaken intramolecular hydrogen bonds, thereby exposing previously hidden groups and promoting partial unfolding of protein molecules. This structural opening may facilitate the formation of covalent bridges between the SPI polypeptide chains and the phenolic rings of *Aronia* anthocyanins (Zhou et al., 2026), as suggested by the appearance of the new aromatic peak at 1443.7 cm^−1^ and shifts in the amide III region. The shift in O—H stretching (3271.3–3269.9 cm^−1^) further confirms that plasma-induced unfolding provides additional accessible sites for hydrogen bonding and van der Waals interactions between the opened protein matrix and the anthocyanins. These modifications suggest that CP treatment increases protein flexibility and susceptibility to unfolding, which may contribute to the enhanced functional properties of SPI-AME complexes (Hou et al., 2025). While both treatments contribute to structural changes, interactions with AME phenolics (e.g., cyanidin-3-*O*-glucoside) appeared to exert a more significant influence on the SPI secondary structure compared to CP treatment alone, particularly through intensified phenolic-protein interactions.

### 3.4 Antioxidant activity before and after digestion

The antioxidant activity (DPPH and CUPRAC) and total phenolic content (TPC) analyses exhibited significant variations across different digestion phases (*p* < 0.05, Table S1). For untreated AME, the DPPH activity increased from 12,362.4 ± 158.5 mg Trolox/100 g d.w in the initial phase to 14,812.2 ± 13.9 mg Trolox/100 g d.w in the gastric phase and then decreased to 7498.8 ± 122.3 mg Trolox/100 g d.w in the intestinal phase, whereas the CUPRAC values followed a similar trend, peaking under gastric conditions. These results suggest that the acidic gastric environment enhances the release and apparent accessibility of antioxidant compounds, resulting in higher measured antioxidant activity. While SPI alone showed minimal antioxidant properties, its complexation with AME, particularly in the SPI-AME CP 1:1 group (*v/v*), indicated better preservation of phenolic compounds during gastric digestion, suggesting that balanced protein-anthocyanin interactions favor stability. The intestinal digestion values generally decreased due to phenolic breakdown; however, in some combinations (e.g., the SPI-AME CP 4:1 and SPI-AME 4:1 groups *(v/v)*), TPC increases after digestion were noted, suggesting that protein–phenolic complexes may modulate phenolic availability during digestion.

Compared with their untreated counterparts, CP-treated samples presented different antioxidant profiles. AME-CP and SPI-AME CP complexes initially had lower CUPRAC and TPC values than those of CP-untreated samples, indicating the degradation or transformation of some phenolic compounds. In contrast, during the gastric and intestinal phases, the DPPH and TPC values of AME CP remained greater than those of AME. In the SPI-AME CP 1:1 group *(v/v)*, the post-digestion TPC (51.2 mg gallic acid/100 g d.w) value was greater than that in the untreated CP group (23.9 mg gallic acid/100 g d.w), indicating that CP might modify protein–phenolic interactions, contributing to improved phenolic stability and retention during digestion. In higher-ratio mixtures (e.g., SPI–AME CP 2:1 and 4:1 *(v/v)*), a similar pattern was observed: an initial reduction in phenolic compounds followed by improved preservation in later digestion phases. These findings are in line with previous studies showing that protein–anthocyanin interactions alter protein secondary structure, potentially promoting anthocyanin release during digestion (Jiang et al., 2019; Ren, Xiong, & Li, 2019; Ren, Xiong, Li, & Li, 2019; Sui et al., 2018). Similar improvements in binding affinity have been noted with heat-treated SPI (Zhang et al., 2018), though excessive binding may eventually limit release (Jiang et al., 2019).

During CP processing, ROS/RNS can disrupt phenolic-protein complexes to facilitate radical-mediated cross-linking and new structural interactions (Nemli et al., 2024). These rearrangements affect both phenolic and protein functionality, influencing antioxidant activity, digestibility, and nutritional value (Nemli et al., 2024). Similarly, Zargarchi and Esatbeyoglu (2024) revealed that CP treatment significantly increased DPPH, ferric-reducing antioxidant power (FRAP), ABTS assays, and total phenolic content, reaching to 100.9 Trolox equivalent (TE) μmol·L^−1^, 8.23 Fe^2+^·g^−1^, 0.293 μmol·g^−1^, and 32.80 gallic acid equivalent (GAE)/g, respectively. Furthermore, plasma-induced release of free phenolic acids from complexes can partially increase TPC in later stages (Yu et al., 2020). In a similar study, the 11S fraction exhibited greater binding affinity to anthocyanins than 7S fractions of heat treated SPIs, supporting the formation of strong complexes that increased the stability of anthocyanins due to the conformational rearrangement of SPI (Dumitrascu et al., 2020). CP-treated plant-based foods also experience an increase in antioxidant components due to the disruption of cell membranes by active plasma species, resulting in the release of bioactive compounds and a membrane-eroding effect (Oner et al., 2023; Zargarchi et al., 2024).

**Fig. 3:**
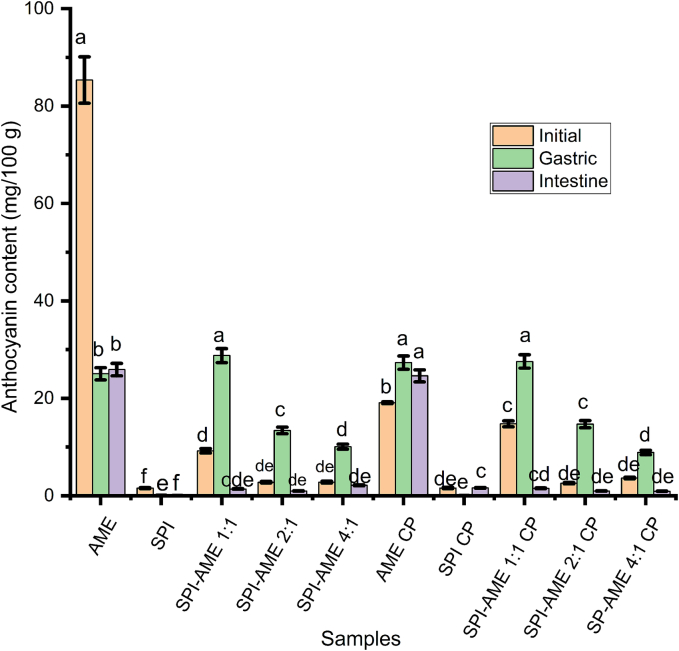
Changes in anthocyanin content (mg/100 g) of *Aronia melanocarpa* extract (AME), soy protein isolate (SPI), and their complexes (*v/v*) before and after cold plasma (CP) treatment during *in vitro* gastric and intestinal digestion phases. Different lowercase letters above the bars indicate significant differences (*p* < 0.05) among treatment groups within the same digestion phase. AME: *Aronia Melanocarpa* Extract, SPI: Soybean protein isolate, CP: Cold plasma, SPI-CP: Cold plasma treated-SPI, SPI-AME 1:1: SPI-AME interaction at 1:1 ratio (*v/v*), SPI-AME CP 1:1: Cold plasma treated-SPI-AME interaction at 1:1 ratio (*v/v*), SPI-AME 2:1: SPI-AME interaction at 2:1 ratio (*v/v*), SPI-AME CP 2:1: Cold plasma treated-SPI-AME interaction at 2:1 ratio (*v/v*), SPI-AME 4:1: SPI-AME interaction at 4:1 ratio (*v/v*), SPI-AME CP 4:1: Cold plasma treated-SPI-AME interaction at 4:1 ratio (*v/v*).

CP treatment significantly influenced the anthocyanin content of SPI-AME complexes during *in vitro* digestion (*p* < 0.05), with notable variations observed across the initial, gastric, and intestinal phases (Fig. 3). The untreated AME presented the highest initial anthocyanin concentration, highlighting its rich bioactive profile. However, this content significantly decreased during digestion, likely due to anthocyanin transformation and degradation under gastrointestinal conditions. While the SPI had the lowest anthocyanin content across all the digestion phases, the incorporation of anthocyanins into the SPI significantly increased the anthocyanin levels in the complexes, particularly in the SPI-AME 1:1 (*v/v*) formulation. This trend suggests that higher anthocyanin concentrations initially contribute to improved anthocyanin stabilization within the protein matrix. The application of CP treatment had a protective effect on anthocyanin stability. Cold-plasma-treated SPI-AME complexes (e.g., SPI-AME 1:1 CP (*v/v*)) presented greater anthocyanin retention across all digestion phases than did untreated samples, which may be due to plasma-induced structural modifications, increasing pigment stability.

CP may enhance stability primarily through hydrogen bonds, hydrophobic interactions, and π–π interactions (Wang, Jia, et al., 2024; Zhang et al., 2023). While passive encapsulation (e.g., β-cyclodextrin, starch, cyclodextrin-metal organic framework (MOFs)) provide a physical shield against light, oxidation, and heat (Chen et al., 2024; Molet-Rodríguez et al., 2024; Xue, Zhao, et al., 2024; Yin et al., 2025), CP applications offer an active modification mechanism. Literature studies have shown that CP oxidatively breaks down phenolic macromolecules into smaller, more biologically active compounds, thereby increasing antioxidant and anticancer activity, and simultaneously improving the crystal structure and thermal stability of the matrix (e.g., starch) (Tang et al., 2023). Our results highlight CP as a dynamic strategy that strengthens protein-phenolic interactions and may contribute to reduced anthocyanin losses during intestinal digestion. Interestingly, SPI-CP alone also slightly improved stability, suggesting that plasma-induced surface modifications may enhance molecular interactions. Collectively, these findings underscore the potential of CP treatment for developing functional foods with improved bioactive delivery and stability profiles.

### 3.5 Determination of phenolic compounds via UHPLC-PDA analysis

UHPLC-PDA results were examined before and after sample digestion, revealing significant differences in the amounts of different phenolic acids (neochlorogenic and chlorogenic acids) and major anthocyanins (cyanidin-3-*O-*galactoside, cyanidin-3-*O*-glucoside, cyanidin-3-*O*-arabinoside, and cyanidin-3-*O*-xyloside), as shown in Table S2.

A sharp increase in the phenolic acid and anthocyanin contents of AME was observed during the gastric digestion phase, whereas anthocyanin levels decreased significantly during the intestinal phase. The high antioxidant capacity (Table S2) observed in the stomach phase of the AME and AME-CP samples was consistent with the phenolic acid and anthocyanin release determined by UHPLC. Furthermore, the decrease in antioxidant capacity observed in the small intestine in these samples is proportional to the decrease in anthocyanin content observed by means of UHPLC. Studies have suggested that this decrease in the intestinal phase is due to the instability of anthocyanins at pH 7.0 and their tendency to undergo rapid degradation (McDougall et al., 2005). In general, complete digestion of anthocyanins does not occur in the gastric environment, however the increase in anthocyanin content observed in the gastric phase may be associated with the acidic environment stabilizing the flavylium cation form and facilitating early absorption via translocase enzyme (Wu et al., 2023). On the other hand, phenolic stability increased in CP-treated samples compared with untreated samples during digestion. Soy protein, whether treated with CP or not, had very low phenolic content, as observed in the spectrophotometric analysis results in Table S1, and was undetectable by UHPLC (Table S2). However, after soy protein interacted with AME at a 1:1 ratio (*v/v*), it contained a higher phenolic content than the other mixing ratios did (2:1 and 4:1 *(v/v)*). The literature has shown that moderate protein–polyphenol interactions contribute more to the bioavailability of polyphenols than does excessive binding (Wu et al., 2023). The CP-treated SPI–AME (1:1, *v/v*) sample showed higher stability during digestion compared to the untreated sample. This finding is consistent with DPPH and CUPRAC results, suggesting that plasma treatment may lead to partial conformational changes in the protein structure, exposing reactive amino acid residues and strengthening protein-polyphenol interactions. These interactions may have enhanced the stability of polyphenols during digestion through non-covalent bonding such as hydrogen bonds and hydrophobic interactions, as well as possible oxidative processes (Ozdal et al., 2013; Ozdal et al., 2019; Pankaj et al., 2018). CP treatment may have contributed to subtle conformational changes and structural reorganization in soy protein without causing extensive denaturation. According to the FTIR analysis, the wavenumber shifts and intensity increases observed, particularly in the amide I and amide II regions, are associated with protein rearrangement, partial denaturation, or structural reorganization. Furthermore, changes in the amide III region suggest oxidative and conformational modifications. In general, CP treatment affects functional behavior by altering a protein's structural properties rather than significantly changing its secondary structure. In the SPI-AME complex, *Aronia* polyphenols appear to contribute to protein stabilization, and shifts in the amide I, II, and III regions in the FTIR spectrum indicate regulatory interactions and structural reorganization of the protein structure. FTIR analysis results indicate that CP treatment induced mild conformational flexibility or reorganization without significantly denaturing the protein. According to the FTIR analysis results, in the presence of AME and CP treated (1:1 ratio *(v/v)*) indicates an increase in the alpha-helical content and a more ordered protein structure. In summary, the structural characteristics of AME appear to promote a more ordered protein conformation, whereas decreasing AME content reduces the structural order of SPI. Compared with AME incorporation, CP treatment exerted a relatively limited effect on protein secondary structure.

### 3.6 Bioaccessibility of phenolic compounds of *Aronia melanocarpa*

As shown in Table S3, a decrease in bioaccessibility occurred during the transition of polyphenols, particularly anthocyanins, from the acidic stomach environment to the more basic intestinal environment. Anthocyanins are unstable at neutral pH values and are known to have difficulty maintaining their stability (Bouayed et al., 2011). The findings in Table S3 indicate that the bioaccessibility of anthocyanins during digestion is strongly shaped by both the components in the food matrix and the processing conditions applied. The greater than 100% increase in bioaccessibility observed in the gastric phase of the AME samples in Table S3 can be explained by the release of bound phenolics from the food matrix under gastric conditions. The pure AME exhibited very high bioaccessibility in the gastric phase (e.g., cyanidin-3-*O-*galactoside), however, reflecting a relative release compared to the initial extractable amount during digestion, rather than an absolute measure of bioavailability. This can be explained by the acidic pH in the stomach environment, which stabilizes the flavylium form of anthocyanins and facilitates early digestion, whereas alkaline conditions in the intestine create structural instability.

The interaction of anthocyanins with proteins offers an effective protective strategy against this instability. Dietary proteins, particularly SPI, stabilize the molecule by complexing with anthocyanins, which may involve hydrogen bonds and hydrophobic interactions. These bindings regulate the gradual release of anthocyanins during digestion and increase their bioaccessibility. CP-treated extract showed greater phenolic preservation in both the gastric and intestinal phases; for example, the intestinal bioaccessibility of cyanidin-3-*O-*glucoside was 1238.3%, compared to AME-CP, whereas it was only 28.9% in AME. Similarly, the SPI–AME complex showed a significant increase in neochlorogenic acid (124.2%) and cyanidin-3-*O-*galactoside (85.9%) in the gastric phase, but this effect diminished in the intestine. The CP-treated SPI–AME (1:1) complex provided more balanced phenolic preservation in both the gastric (89.4%) and intestinal (83.9%) phases. These findings suggest that plasma treatment alters protein structural properties, resulting may enhance interactions with anthocyanins and greater stability in the intestinal environment. Additionally, mixing ratios stand out as an important parameter; the SPI–AME CP complex at a 2:1 ratio exhibited 88.8% intestinal bioaccessibility for neochlorogenic acid, yielding better results than the 1:1 ratio. However, the presence of excess protein at a 4:1 ratio may contribute to excessive binding of polyphenols, inhibiting post-digestive release. In conclusion, these findings indicate that the bioaccessibility of anthocyanins becomes significant when evaluated in conjunction with factors such as the pH of the gastrointestinal environment, the type and intensity of protein–phenolic interactions, processing conditions, and the mixing ratio.

Some studies have reported an increase in phenolic content after simulated *in vitro* digestion, whereas others have reported a decrease (Zeng et al., 2019). The food matrix, pH, digestive enzymes, and other factors determine the release and stability of phenolics (Bouayed et al., 2011). It has been determined that some phenolic acids, such as chlorogenic acid, are bound to cell wall components or proteins via ester bonds and that these bonds are hydrolyzed in the acidic environment of the stomach, rendering the phenolics soluble (Clifford, 2000). Interactions with soy protein and CP treatment of the samples resulted in changes in the matrix structure. Studies indicate that hydrogen bonding, hydrophobic interactions, van der Waals forces, and π–π stacking collectively contribute to the stability of anthocyanin–protein/peptide complexes, with no single interaction acting as the sole dominant force (Li et al., 2025; Liao et al., 2021; Xue et al., 2024; Zang et al., 2022). In particular, π–π interactions play an important but cooperative role alongside other non-covalent interactions in maintaining overall stability. Moreover, the combined effect of multiple weak interactions has been reported to enhance complex stability. However, there is no consistent quantitative evidence demonstrating that π–π interactions are systematically stronger than hydrophobic interactions in these systems (Wang et al., 2024). Therefore, selecting appropriate protein types and processing conditions is critical for improving anthocyanin stability and enabling controlled release during digestion (Ma et al., 2022).

### 3.7 *In silico* evaluation of interactions between soy protein and *Aronia melanocarpa* extracts

Molecular docking analysis between the ligands and 1FXZ was performed to determine the binding potentials of the ligands to glycinin. The results obtained are summarized in Table 3 including interaction maps, binding energies (BE, kcal.mol^−1^), interaction types and distances (Å). As known from the literature, binding is more likely to occur when the binding energy is less than −6 kcal.mol^−1^ (Zhao et al., 2022). Table 3 shows that each ligand has the potential to bind to glycinin.

**Table 3:**
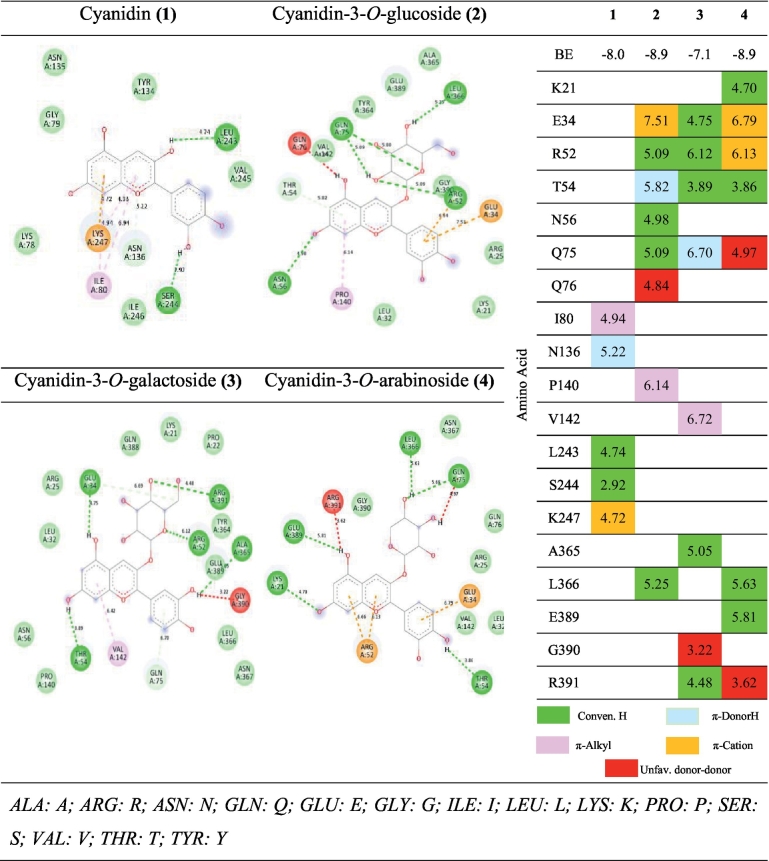
Binding energies (BE, kcal.mol^−1^), interaction types and distances (Å) between ligands and binding site amino acids of SPI.

Cyanidin-3-*O*-glucoside and cyanidin-3-*O*-arabinoside have the highest binding affinity with −8.9 kcal.mol^−1^; this difference in their binding affinities compared to cyanidin and cyanidin-3-*O*-galactoside theoretically indicates an increased binding potential. This stable increase in binding energy suggests that cyanidin-3-*O*-glucoside and cyanidin-3-*O*-arabinoside may offer a higher binding affinity (Kuntz et al., 1999). Compared with the other compounds, cyanidin-3-*O*-galactoside had a low affinity for 1FXZ. The active amino acids were GLU34, ARG52, THR54 and GLN75 when anthocyanidin-3-*O*-glycosides were docked to 1FXZ, which means that the carbohydrate moieties at the C3 position had binding characteristics to those residues. Hydrogen-bond interactions were observed between cyanidin-3-O-galactoside, cyanidin-3-O-arabinoside, and THR54, with bond lengths of 3.89 and 3.86 Å, respectively. Furthermore, cyanidin-3-*O*-glucoside had the most interactions among the interaction types, but the bond lengths were mostly longer than those of cyanidin-3-*O-*arabinoside. The results suggested that the connection between them and 1FXZ takes place via conventional hydrogen bonds and unfavorable donor–donor interactions with small distances of less than ∼6 Å. In addition, the strong unfavorable donor–donor interaction (3.22 Å) may be the reason for the low binding affinity of cyanidin-3-*O*-galactoside. Literature molecular modeling analyses also support these findings, which revealed that glycine and SPI have high binding energies with anthocyanins, particularly cyanidin-3-*O-*glucoside and cyanidin-3-*O-*rutinoside. Changes in the secondary structural elements of proteins (e.g., increased α-helix ratios and decreased hydrogen bonding) following heat treatment create new interaction sites that facilitate anthocyanin binding (Dumitrascu et al., 2020).

When the findings obtained from molecular docking results are evaluated together with the experimental results, the reason for the increased stability in binding affinity becomes more significant. Specifically, according to FTIR analysis, the CP treatment resulted in rearrangements in the conformation of SPI, increased flexibility in the existing structure, and the emergence of additional binding sites (Table 2, Fig. S1 and S2). This structural change may contribute to the stronger binding affinity predicted for cyanidin-3-O-glucoside and cyanidin-3-O-arabinoside toward SPI (Table 3). It was determined that the stable binding affinity in the relevant compounds arises from the conventional hydrogen bonds between the amino acids listed in Table 3 and their -OH groups, and via π–π interactions between aromatic rings, which also explains their stronger interactions with SPI compared to other anthocyanins (Dumitrascu et al., 2020; Wang et al., 2024; Xue et al., 2024). For example, cyanidin-3-*O*-galactoside and cyanidin-3-*O*-arabinoside, determined as 3.89 and 3.86 Å respectively, given in Table 3, are provided to support the higher stability in SPI-AME complexes. These stable complexes formed showed better retention and stability of anthocyanins during gastrointestinal digestion (Fig. 3, Table S3). This indicates that enhanced protein–phenolic interaction plays a key role in protecting anthocyanins from degradation and regulating their release (Ozdal et al., 2013; Wu et al., 2023). Therefore, the combined assessment of FTIR, digestion experiments, and docking analysis suggests that CP-induced structural modifications may improve binding capacity, thereby enhancing anthocyanin stability and bioaccessibility. Molecular docking simulations provide theoretical predictions of potential interaction patterns; however, these primarily identify possible non-covalent interactions and cannot fully supported binding mechanisms under plasma-treated or gastrointestinal digestion conditions. Therefore, docking results should be interpreted as supporting computational evidence rather than definitive proof of interaction mechanisms. These strong interactions likely promote the retention of anthocyanins within the protein matrix, reducing their direct exposure to environmental stressors such as pH changes and digestive enzymes. Consequently, anthocyanins are partially conserved in the gastric phase and released in a more controlled manner in the intestinal phase. This controlled release behavior explains the improved stability and bioavailability observed in CP-treated SPI–AME systems.

## 4 Conclusion and future remarks

This study presents a comprehensive evaluation of the effects of CP technology and complexing with AME on the structural and digestive properties of SPI. FTIR analyses showed that CP treatment (80 W, 10 min) increased random coil content in SPI, suggesting enhanced conformational flexibility, whereas AME addition promoted a more ordered structure through increased α-helix content. Spectrophotometric and UHPLC-PDA analyses suggested that CP treatment significantly increased the bioaccessibility of anthocyanins in the gastric and intestinal phases compared to samples without plasma treatment. In addition, *in silico* docking analyses supported the formation of hydrogen bonds between cyanidin-3-O-glucoside, cyanidin-3-O-arabinoside, and the THR54 residue of 1FXZ.

While providing a framework for understanding the structural-functional modifications in SPI-AME systems, this study primarily focuses on interaction trends and digestion-related behavior rather than providing definitive biochemical evidence for specific covalent interaction pathways**.** In spite of the extensive studies on protein- anthocyanin interactions, optimization of these systems remains a challenge. Anthocyanins with different chemical structures may exhibit different binding affinities with proteins; therefore, the binding mechanisms of specific anthocyanins require detailed investigation. Future studies should prioritize the identification of proteins with favorable surface properties, self-assembly behavior, and gel-forming capacity to optimize anthocyanin entrapment within protein matrices and enhance their stability throughout gastrointestinal digestion.

Furthermore, as a non-thermal technology, CP has been reported to contribute to microorganism and enzyme inactivation, enhancement of antioxidant activity, and preservation of bioactive compounds. In conclusion, the synergy of CP modification and protein-phenolic interactions offers a promising strategy for the protection of sensitive bioactive compounds in the digestive system. These findings provide valuable insights for the optimization of protein-based carrier systems in the development of functional food formulations.

The current work leaves biological efficacy and cellular mechanisms yet to be explored. In particular, the biological activity of AME-SPI complexes should be further evaluated through their antiproliferative and apoptotic effects in colon cancer cell lines. Moreover, the regulatory effects of these systems on key signaling pathways associated with oxidative stress and inflammation, such as NF-κB and PI3K/Akt, should be investigated at the molecular level. In addition, metabolomic approaches are proposed for identifying bioavailable phenolic compounds after digestion and elucidating their effects on cellular metabolism. Interaction with the gut microbiota and potential prebiotic effects also constitute an important area of research. Furthermore, validation of protein-phenolic interaction mechanisms through advanced structural analyses and optimization of cold plasma parameters will enhance the scientific depth of the study. Finally, validation of biological efficacy with *in vivo* models and evaluation of the applicability of the developed system to functional foods or nutraceutical products will reveal the translational potential of this approach.

## CRediT authorship contribution statement

**Büşra Yusufoğlu:** Writing – original draft, Methodology, Investigation, Formal analysis, Data curation. **Rukiye Gundogan:** Writing – original draft, Methodology, Investigation, Formal analysis. **Yeşim Ayık:** Writing – original draft, Methodology. **Oliver Görlitzer:** Methodology. **Vildan Enisoğlu Atalay:** Writing – review & editing, Methodology. **Esra Capanoglu:** Writing – review & editing. **Tuba Esatbeyoglu:** Writing – review & editing, Supervision, Resources, Project administration, Funding acquisition, Conceptualization.

## Funding

The publication of this article was funded by the Open Access Fund of the Leibniz Universität Hannover, Germany.

## Declaration of competing interest

The authors declare the following financial interests/personal relationships which may be considered as potential competing interests: Given her role as Editor, Tuba Esatbeyoglu, had no involvement in the peer-review of this article and has no access to information regarding its peer-review.

## Data Availability

Data will be made available on request.

## Appendix A Supplementary data

Supplementary material Supplementary Data: FTIR spectrums of Cold Plasma-Treated vs. Untreated Soy Protein–Anthocyanin Complexes (Fig. S1), Second derivative and spectral deconvolution analysis of a FTIR spectrums of Cold Plasma-Treated vs. Untreated Soy Protein–Anthocyanin Complexes (Fig. S2), Total phenolic content (TPC), the DPPH radical scavenging activity, the Cupric reducing antioxidant capacity (CUPRAC) in Cold Plasma-Treated vs. Untreated Soy Protein–Anthocyanin Complexes after in vitro digestion (sS1), Quantification by UPLC-PDA as equivalents of the standards used (Table S2), Relative bioaccessibility (%) of individual phenolic compounds in gastric and intestinal phases (Table S3).
