# Supplementary material for: Effect of soybean protein isolate-Aronia melanocarpa extract interaction on the bioaccessibility of anthocyanins after low-pressure cold plasma treatment
Source: Food Chem X. 2026 May 18;37:104000. doi: 10.1016/j.fochx.2026.104000 (PMC13292393; doi:10.1016/j.fochx.2026.104000)
Supplement: Supplementary file 1 — Supplementary material Supplementary Data: FTIR spectrums of Cold Plasma-Treated vs. Untreated Soy Protein–Anthocyanin Complexes (Fig. S1), Second derivative and spectral deconvolution analysis of a FTIR spectrums of Cold Plasma-Treated vs. Untreated Soy Protein–Anthocyanin Complexes (Fig. S2), Total phenolic content (TPC), the DPPH radical scavenging activity, the Cupric reducing antioxidant capacity (CUPRAC) in Cold Plasma-Treated vs. Untreated Soy Protein–Anthocyanin Complexes after in vitro digestion (sS1), Quantification by UPLC-PDA as equivalents of the standards used (Table S2), Relative bioaccessibility (%) of individual phenolic compounds in gastric and intestinal phases (Table S3). [file mmc1.docx]

SUPPLEMANTARY MATERIAL (S)

a


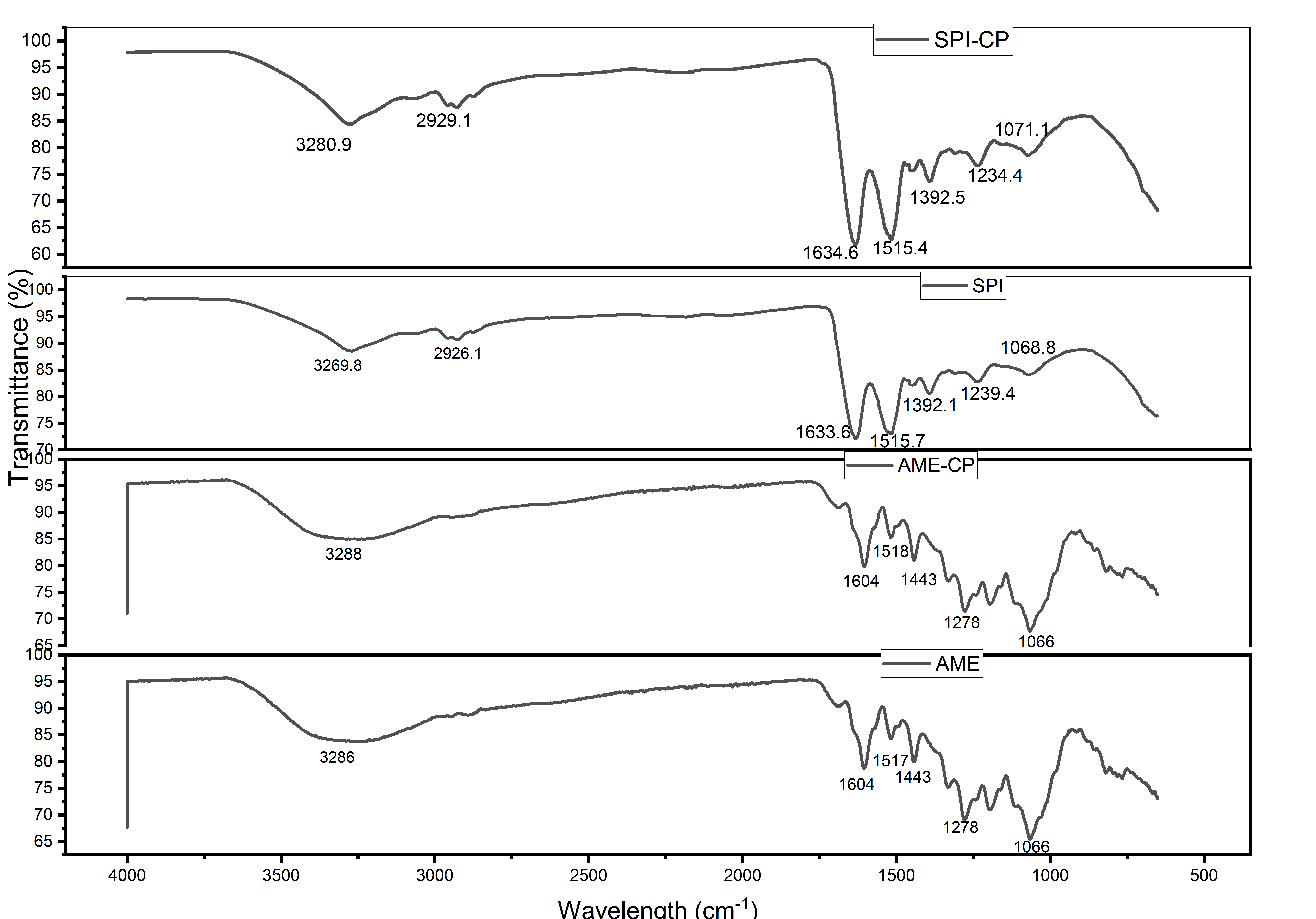


b


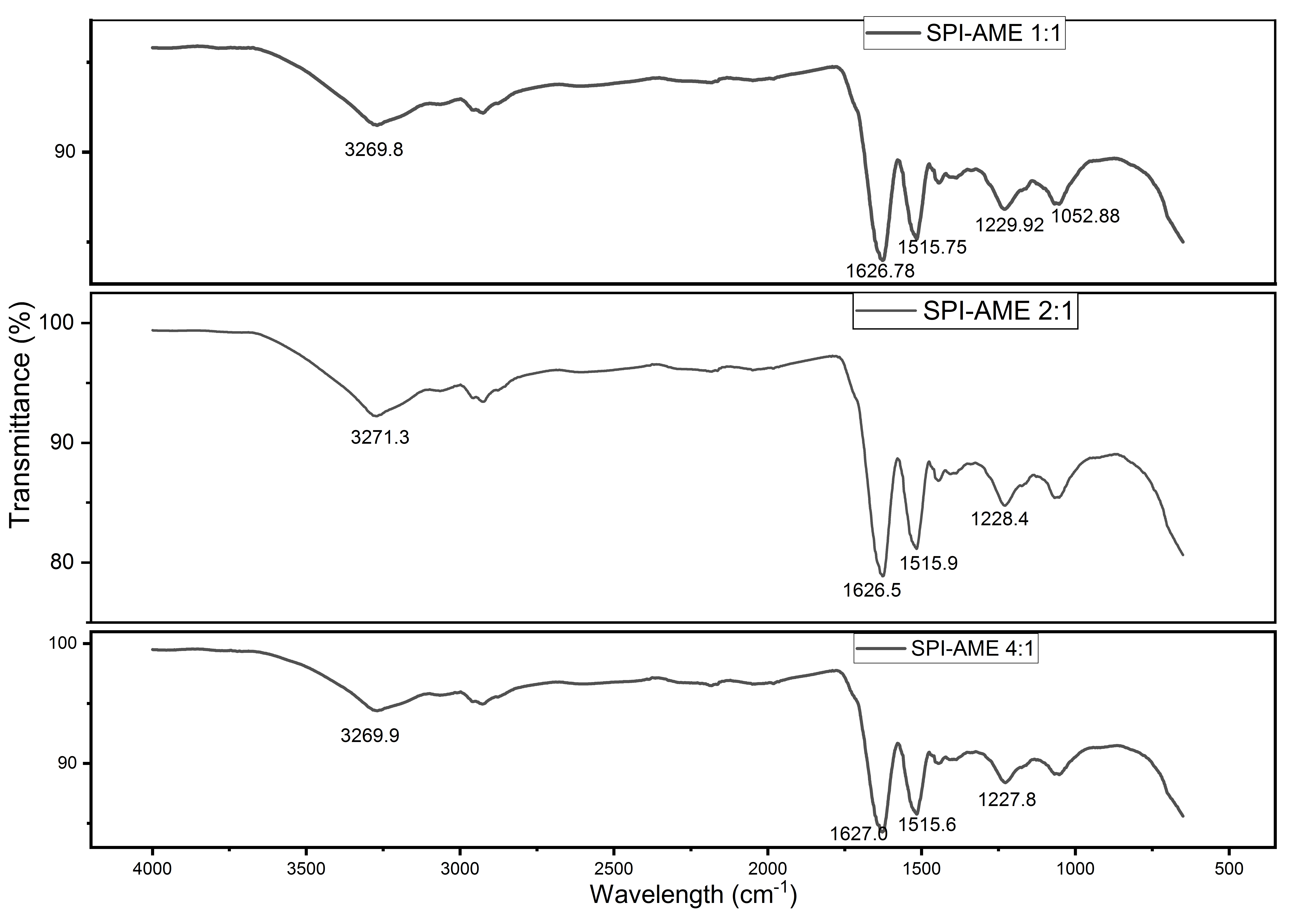


c
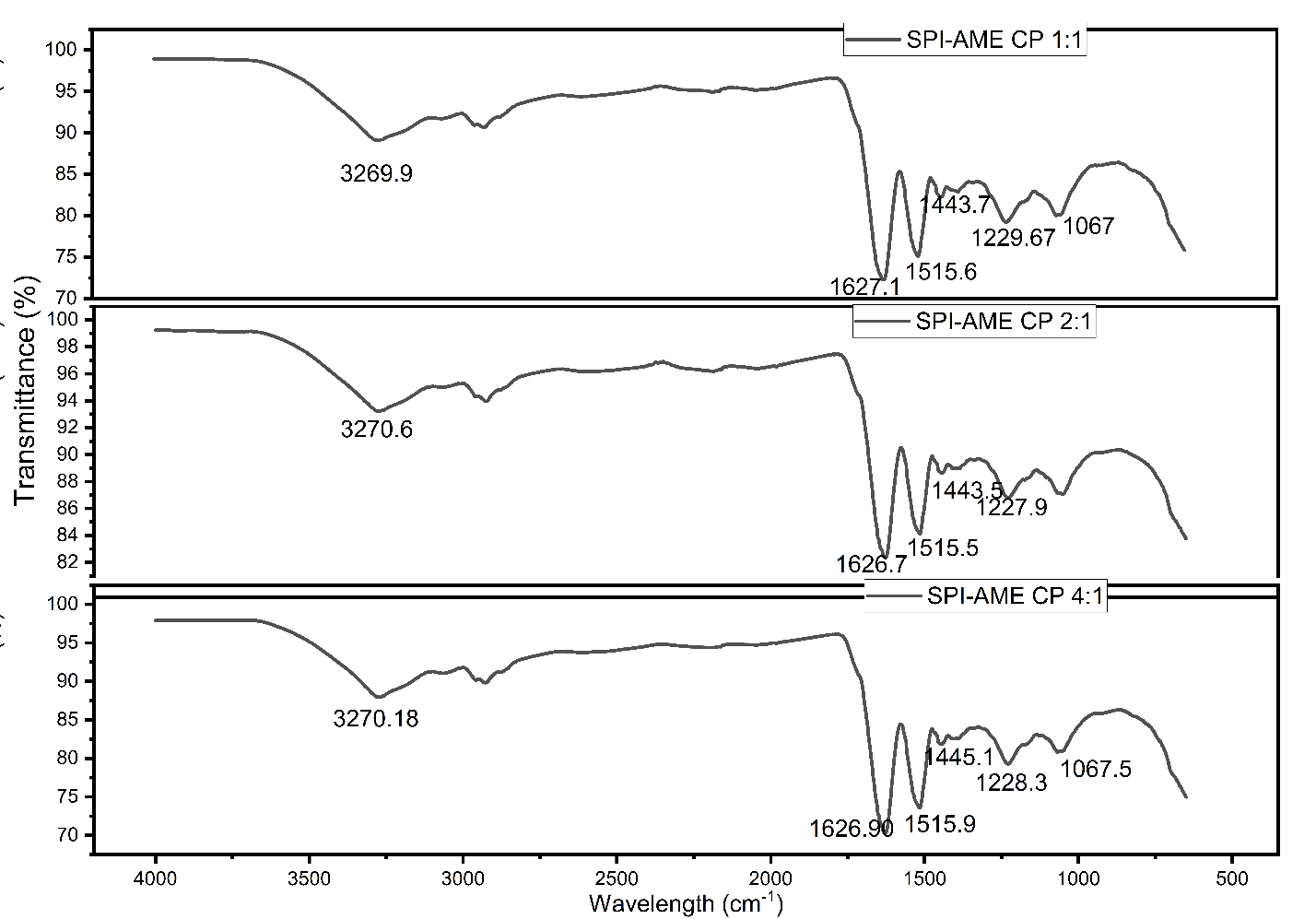


Fig.S1. FTIR spectrums of Cold Plasma-Treated vs. Untreated Soy Protein–Anthocyanin Complexes: Soy Protein Isolate (SPI), Cold plasma treated-SPI, Aronia extract (AME), Aronia extract -CP treated (AME CP)(a), SPI-AME 1:1: SPI-AME interaction at 1:1 ratio (*v/v*), SPI-AME 2:1: SPI-AME interaction at 2:1 ratio (*v/v*), SPI-AME 4:1: SPI-AME interaction at 4:1 ratio (*v/v*) (b), SPI-AME CP 1:1: Cold plasma treated-SPI-AME interaction at 1:1 ratio (*v/v*), SPI-AME CP 2:1: Cold plasma treated-SPI-AME interaction at 2:1 ratio (*v/v*), SPI-AME CP 4:1: Cold plasma treated-SPI-AME interaction at 4:1 ratio (*v/v*) (c).


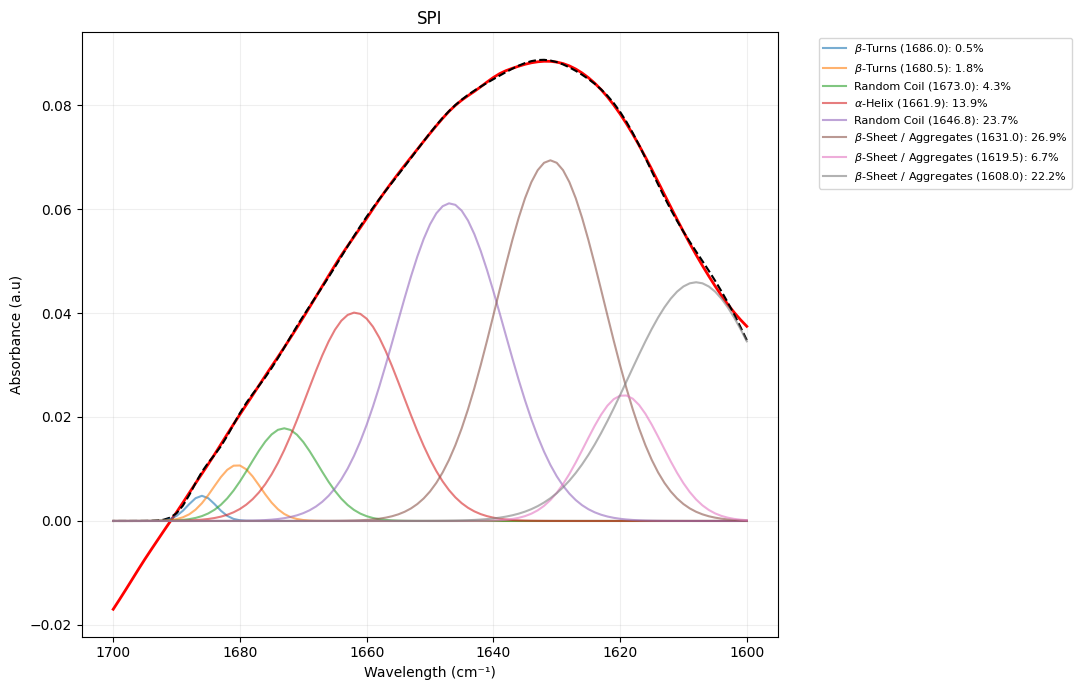

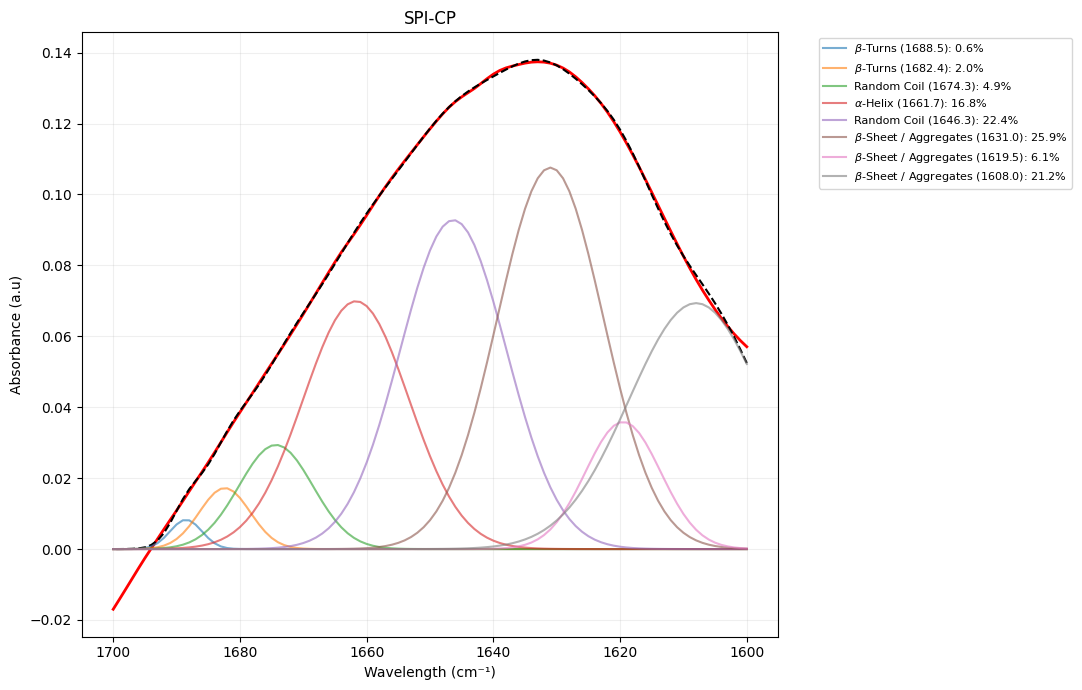

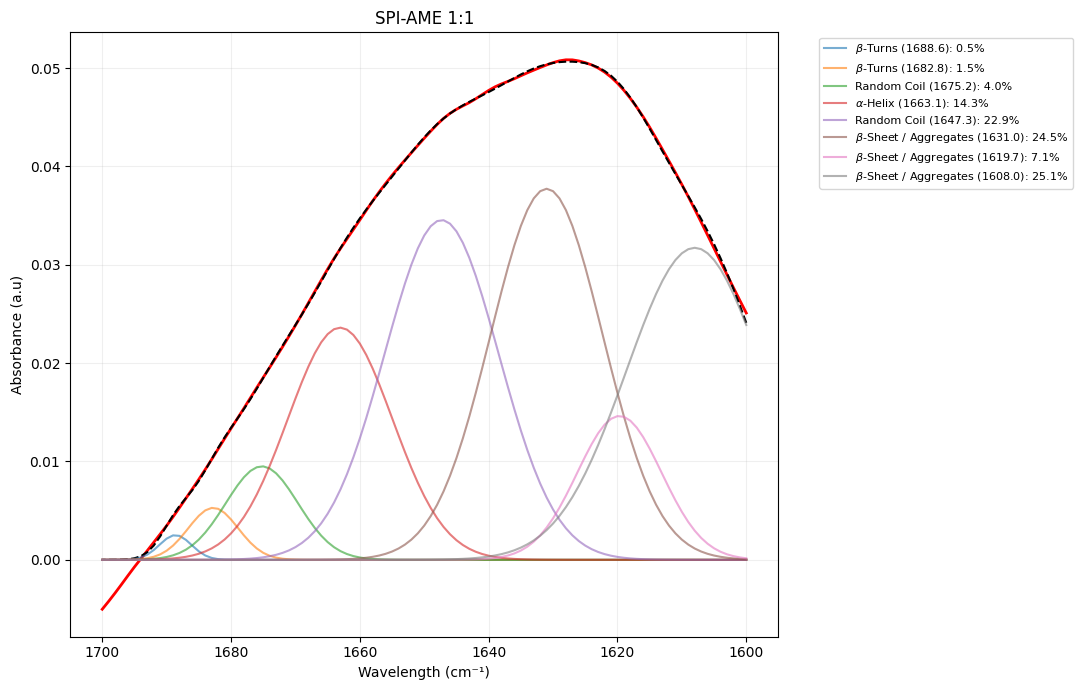

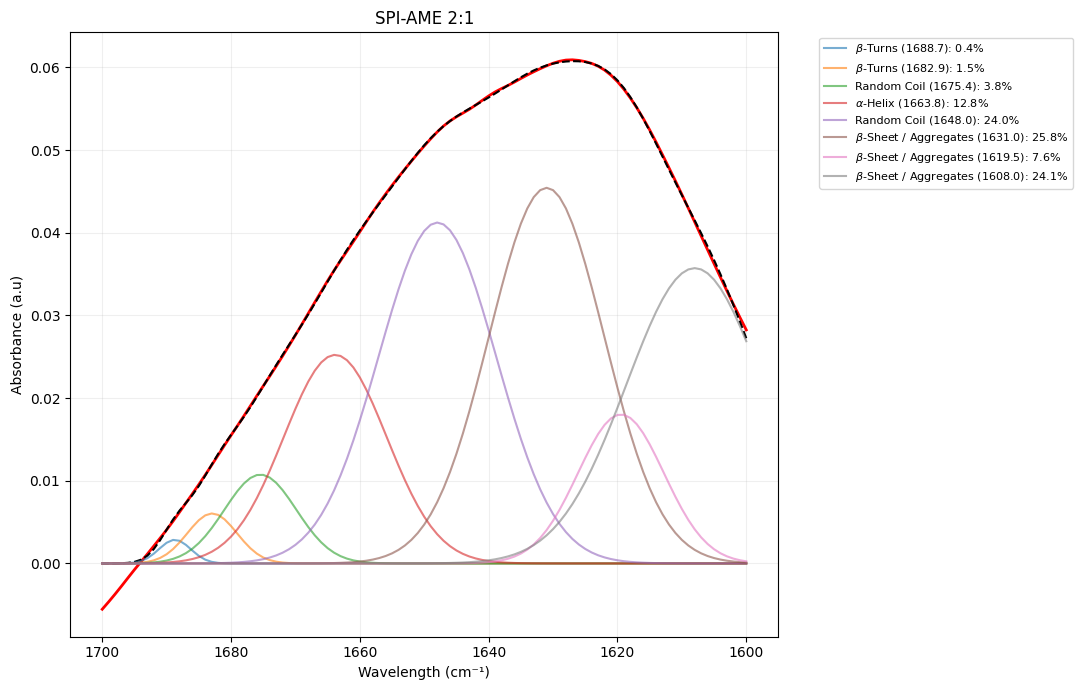

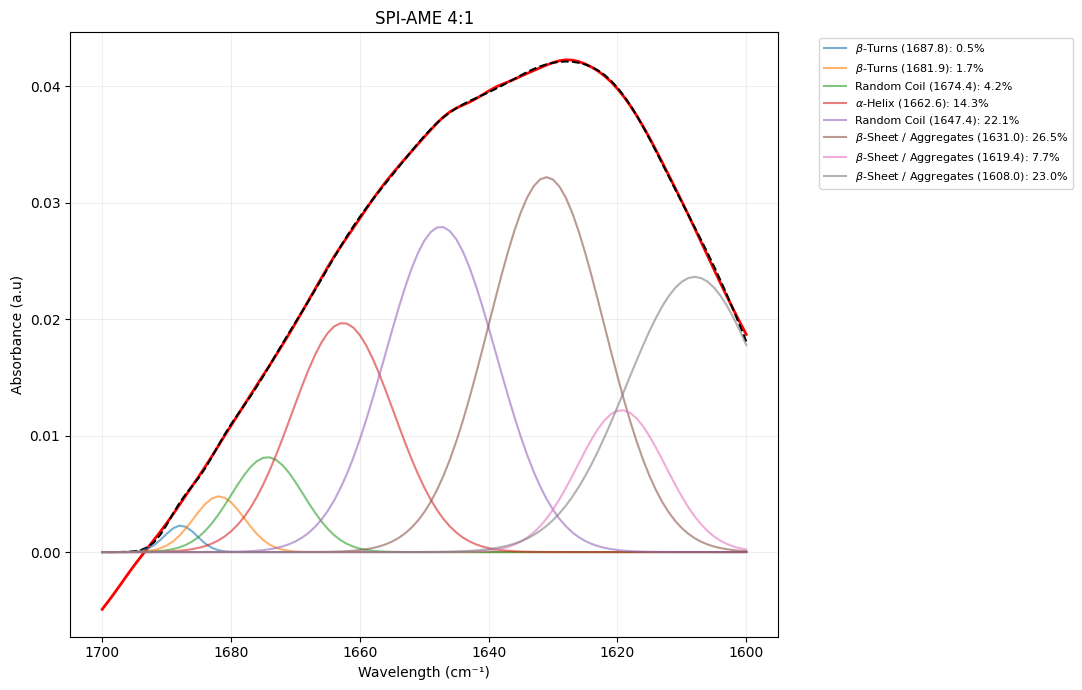


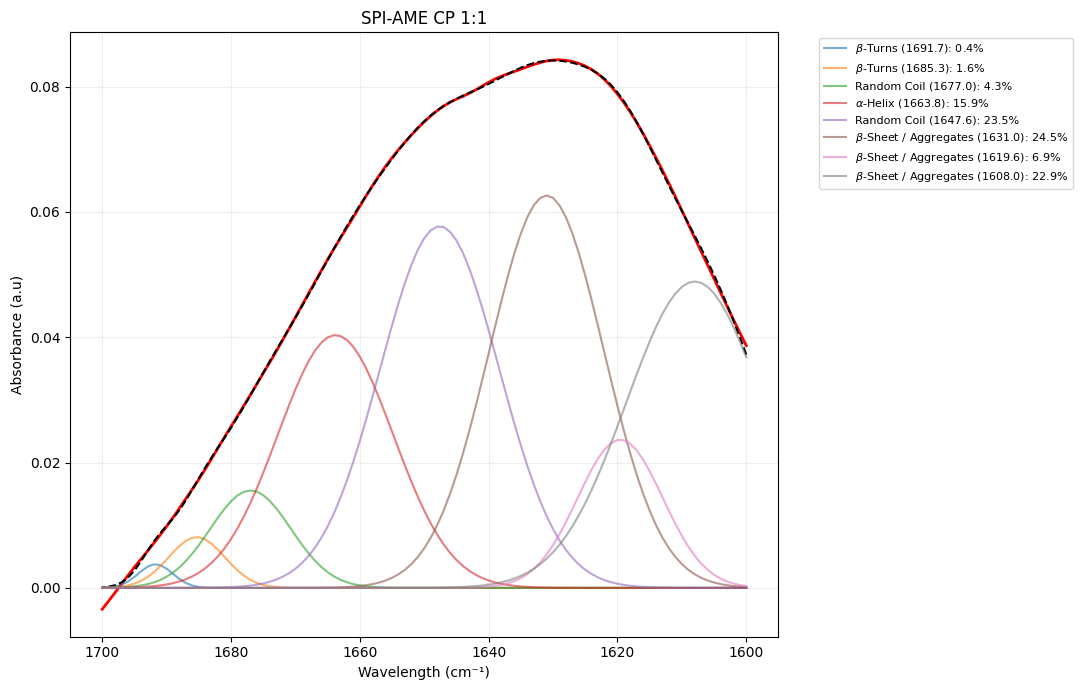


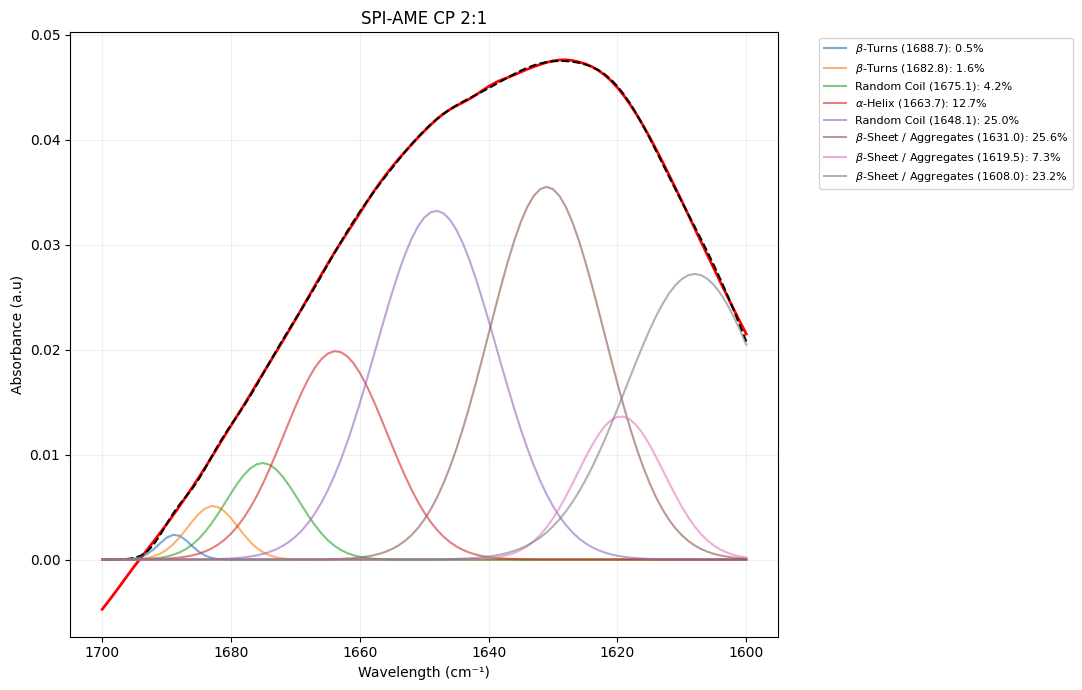

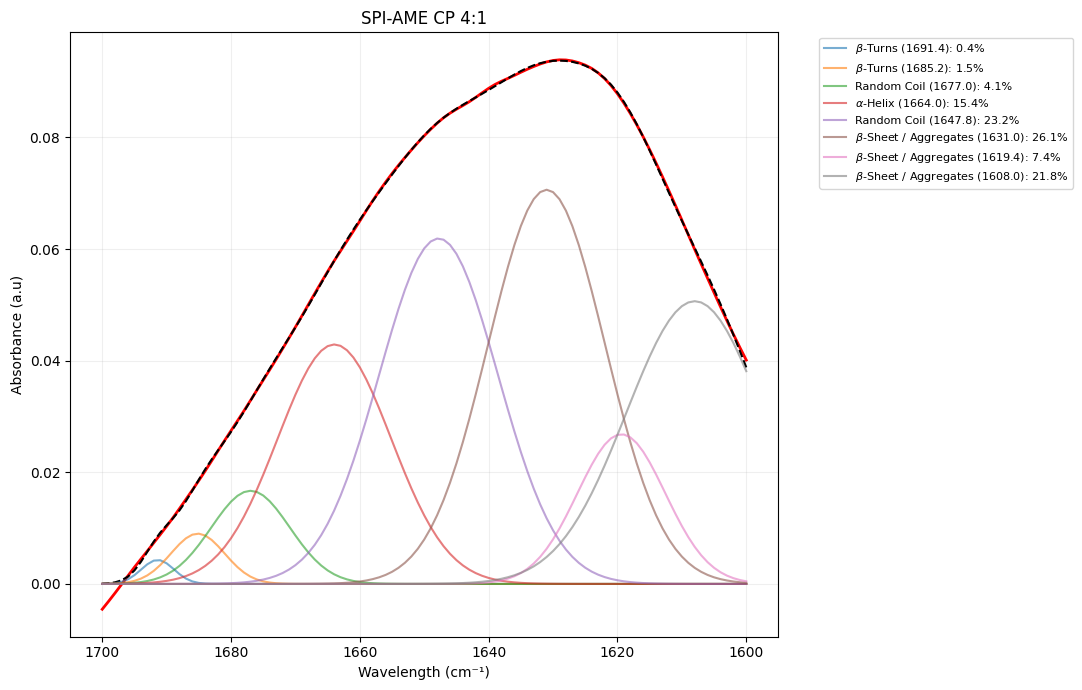


Fig. S2. Second derivative and spectral deconvolution analysis of a FTIR spectrums of Cold Plasma-Treated vs. Untreated Soy Protein–Anthocyanin Complexes: Soy Protein Isolate (SPI), Cold plasma treated-SPI, Aronia extract (AME), Aronia extract -CP treated (AME CP), SPI-AME 1:1: SPI-AME interaction at 1:1 ratio (*v/v*), SPI-AME 2:1: SPI-AME interaction at 2:1 ratio (*v/v*), SPI-AME 4:1: SPI-AME interaction at 4:1 ratio (*v/v*), SPI-AME CP 1:1: Cold plasma treated-SPI-AME interaction at 1:1 ratio (*v/v*), SPI-AME CP 2:1: Cold plasma treated-SPI-AME interaction at 2:1 ratio (*v/v*), SPI-AME CP 4:1: Cold plasma treated-SPI-AME interaction at 4:1 ratio (*v/v*). The experimental data are well fitted by the sum (red continuous curve) of single Gaussian functions (black dashed curves).

| Table S1. Total phenolic content (TPC), the DPPH radical scavenging activity, the Cupric reducing antioxidant capacity (CUPRAC) in Cold Plasma-Treated vs. Untreated Soy Protein–Anthocyanin Complexes after *in vitro* digestion. | | | | | | | | | |
| --- | --- | --- | --- | --- | --- | --- | --- | --- | --- |
| **Samples** | **CUPRAC (mg trolox/100 g sample)** | | | **DPPH (mg trolox/100 g sample)** | | | **TPC (mg gallic acid/100 g sample)** | | |
|  | Initial | Gastric | Intestine | Initial | Gastric | Intestine | Initial | Gastric | Intestine |
| AME | 12362.4± 158.5 ^a^ | 14812±13.9 ^a^ | 7498.8±122.3 ^a^ | 66.3± 6.3^b^ | 125.1±1.5^a^ | 21.3±2.3^b^ | 12.9±0.8^b^ | 50.9±0.7^bc^ | 15.0±0.60^d^ |
| AME CP | 9833±293.4 ^b^ | 11765.3±84.3 ^b^ | 4695.7±84.4 ^b^ | 82.3± 16.2^a^ | 79.5±27.1^b^ | 30.5±2.8^a^ | 20.7±0.4^a^ | 48.6±1.2^c^ | 23.7±0.30^d^ |
| SPI | 9.3±0.6 ^f^ | 5.8±0.3 ^f^ | 6.4±0.4 ^h^ | 0.076± 0.005^c^ | 0.021±0.004^c^ | 0.004±0.007^c^ | 0.0754±0.002^c^ | 0.06±0.003^f^ | 0.11±0.004^e^ |
| SPI-CP | 9.2±0.9 ^f^ | 5.9±0.3 ^f^ | 10.4±0.1 ^g^ | 0.076±0.001^c^ | 0.006±0.003^c^ | 0.04±0.005^c^ | 0.07±0.002^c^ | 0.11±0.002^f^ | 0.002±0.00^e^ |
| SPI-AME 1:1 | 11551.1±161.4 ^a^ | 13196.6±97.5 ^a^ | 2925.7±103.9 ^c^ | 1.3± 0.1^c^ | 2.22±0.17^c^ | 0.08±0.01^c^ | 0.6±0.01^c^ | 56.2±0.6 ^ab^ | 23.9±0.900^c^ |
| SPI-AME CP 1:1 | 397.9±2.3 ^c^ | 405.2±0.9 ^c^ | 118.9±8.3 ^d^ | 1.23±0.06^c^ | 2.34 ±0.5^c^ | 1.5±0.4^c^ | 0.6±0.03^c^ | 60.3±2.1^a^ | 51.2±4.300^b^ |
| SPI-AME 2:1 | 222.2±45.9 ^d^ | 293.2±1.7 ^d^ | 76.9±4.2 ^e^ | 0.19±0.02^c^ | 1.02±0.01^c^ | 0.22±0.04^c^ | 0.3±0.02^c^ | 40.9±2.9^d^ | 44.9±1.100^b^ |
| SPI-AME CP 2:1 | 191.8±45.1 ^d^ | 292.1±5.1 ^d^ | 110.5±2.9 ^d^ | 0.42±0.04^c^ | 0.89±0.009^c^ | 0.05±0.08^c^ | 0.3±0.02^c^ | 40.0±1.0^d^ | 31.0±5.400^c^ |
| SPI-AME 4:1 | 203.6±18.8 ^d^ | 277.6±3.6 ^d^ | 82.7±3.6 ^e^ | 0.16± 0.04^c^ | 0.20±0.05^c^ | 0.27±0.01^c^ | 0.2±0.01^c^ | 53.8±0.2^bc^ | 71.2±1.200^a^ |
| SPI-AME CP 4:1 | 98.3±23.3 ^e^ | 153.2±1.4 ^e^ | 64.2±0.6 ^f^ | 0.29±0.1^c^ | 0.19±0.019^c^ | 0.2±0.03^c^ | 0.2±0.01^c^ | 29.4±1.8^e^ | 28.4±0.900^c^ |
| AME: *Aronia Melanocarpa* Extract, SPI: Soy Protein Isolate, CP:Cold plasma, SPI-CP: Cold plasma treated-SPI, SPI-AME 1:1: SPI-AME interaction at 1:1 *(v/v)*, SPI-AME CP 1:1: Cold plasma treated-SPI-AME interaction at 1:1 ratio *(v/v)*, SPI-AME 2:1: SPI-AME interaction at 2:1 ratio *(v/v)*, SPI-AME CP 2:1: Cold plasma treated-SPI-AME interaction at 2:1 ratio *(v/v)*, SPI-AME 4:1: SPI-AME interaction at 4:1 ratio *(v/v)*, SPI-AME CP 4:1: Cold plasma treated-SPI-AME interaction at 4:1 ratio *(v/v)*.  CUPRAC:2,2-Diphenyl-1-picrylhydrazyl, DPPH: 2,2-Diphenyl-1-picrylhydrazyl, TPC: Total Phenolic Content, Data represent a mean ± SD (n = 3); Mean values represented by the same letter among the dots do not show significant differences (p < 0.05). | | | | | | | | | |

| Table S2. Quantification by UPLC-PDA as equivalents of the standards used. | | | | | | | | | | | | | | | | | | | |
| --- | --- | --- | --- | --- | --- | --- | --- | --- | --- | --- | --- | --- | --- | --- | --- | --- | --- | --- | --- |
| Sample Name | Neochlorogenic acid [mg/L] | | | Chlorogenic acid [mg/L] | | | Cyanidin-3-O-galactoside [mg/L] | | | Cyanidin-3-O-glucoside [mg/L] | | | Cyanidin-3-O-arabinoside [mg/L] | | | Cyanidin-3-O-xyloside [mg/L] | | | |
|  | Initial | Gastric | Intestine | Initial | Gastric | Intestine | Initial | Gastric | Intestine | Initial | Gastric | Intestine | Initial | Gastric | Intestine | Initial | Gastric | Intestine |  |
| AME | 27.30±0.2 | 412.21±2.6 | 116.89±3.5 | 17.39±1.3 | 309.75±2.4 | 92.67±1.5 | 45.17±1.3 | 468.74±2.4 | 13.74±1.6 | 2.28±0.13 | 23.23±0.87 | 0.66±0.01 | 17.61±1.4 | 185.02±5.8 | 7.56±0.58 | 2.82±0.13 | 30.36±2.7 | 1.13±0.23 |  |
| AME-CP | 15.13±1.3 | 416.20±5.7 | 420.27±5.4 | 12.46±0.4 | 297.76±3.7 | 304.56±7.9 | 39.44±1.4 | 467.42±6.5 | 474.52±7.4 | 1.88±0.74 | 22.27±2.6 | 23.28±1.43 | 16.35±1.4 | 182.03±5.6 | 186.54±2.5 | 2.47±0.34 | 29.37±1.5 | 30.77±0.22 |  |
| SPI | U.L. | U.L. | U.L. | U.L. | U.L. | U.L. | U.L. | U.L. | U.L. | U.L. | U.L. | U.L. | U.L. | U.L. | U.L. | U.L. | U.L. | U.L. |  |
| SPI-CP | U.L. | U.L. | U.L. | U.L. | U.L. | U.L. | U.L. | U.L. | U.L. | U.L. | U.L. | U.L. | U.L. | U.L. | U.L. | U.L. | U.L. | U.L. |  |
| SPI-AME 1:1 | 15.3±0.15 | 19.01±1.3 | 5.22±0.45 | 10.6±0.1 | 13.80±0.75 | 4.43±0.02 | 29.0±0.1 | 24.91± 0.8 | 2.16±0.05 | U.L. | 1.20±0.01 | U.L. | 13.0 ±0.0 | 8.93±0.05 | 0.98±0.002 | 7.6±0.1 | 1.34±0.01 | U.L. |  |
| SPI-AME CP 1:1 | 17.0±0.15 | 18.17±0.23 | 18.33±0.25 | 11.6±0.1 | 12.83±1.1 | 12.94±0.87 | 30.6±0.2 | 27.36±0.78 | 25.49±1.2 | U.L. | 1.24±0.02 | 1.24±0.01 | 14.3 ±0.1 | 10.03±0.1 | 9.27±0.03 | 7.3±0.1 | 1.51±0.01 | 1.40±0.01 |  |
| SPI-AME 2:1 | 6.0±0.0 | 8.59±0.03 | 8.72±0.05 | U.L. | 6.12±0.03 | 6.23±0.04 | 12.0±0.4 | 12.97±0.76 | 12.04±0.94 | U.L. | 0.65±0.03 | U.L. | U.L. | 4.69±0.015 | 4.33±0.02 | U.L. | 0.69±0.01 | U.L. |  |
| SPI-AME CP 2:1 | 5.6±0.0 | 18.33±0.1 | 4.97±0.02 | U.L. | 12.94±0.4 | 3.75±0.05 | 14.0±0.2 | 25.49±0.3 | 7.95±0.05 | U.L. | 1.24±0.04 | U.L. | U.L. | 9.27±0.05 | 2.87±0.01 | U.L. | 1.40±0.01 | U.L. |  |
| SPI-AME 4:1 | U.L. | 4.95±0.02 | 4.97±0.02 | U.L. | 3.72±0.01 | 3.75±0.0 | 5.0±0.0 | 9.10±0.02 | 7.95±0.02 | U.L. | 0.40±0.01 | U.L. | U.L. | 3.31±0.01 | 2.87±0.01 | U.L. | 0.43±0.01 | U.L. |  |
| SPI-AME CP 4:1 | U.L. | 4.19±0.02 | 1.08±0.0 | U.L. | 3.11±0.01 | 1.01±0.0 | 3.0±0.0 | 6.99±0.02 | U.L. | U.L. | U.L. | U.L. | U.L. | 2.53±0.03 | U.L. | U.L. | U.L. | U.L. |  |
| AME: *Aronia Melanocarpa* Extract, SPI: Soy Protein Isolate, CP: Cold plasma, AME CP : Cold plasma treated-AME, SPI-CP : Cold plasma treated-SPI, SPI-AME 1:1: SPI-AME interaction at 1:1 ratio *(v/v)*, SPI-AME 2:1: SPI-AME interaction at 2:1 ratio *(v/v)*, SPI-AME 4:1: SPI-AME interaction at 4:1 ratio *(v/v)*, SPI-AME CP 1:1: Cold plasma treated-SPI-AME interaction at 1:1 ratio *(v/v)*, SPI-AME CP 2:1: Cold plasma treated-SPI-AME interaction at 2:1 ratio *(v/v)*, AME CP 4:1: Cold plasma treated-SPI-AME interaction at 4:1 ratio *(v/v)*, U.L.: Under LOD, Data represent a mean ± SD (n = 3). | | | | | | | | | | | | | | | | | | | |

| Table S3. Relative bioaccessibility (%) of individual phenolic compounds in gastric and intestinal phases | | | | | | | | | | | | |
| --- | --- | --- | --- | --- | --- | --- | --- | --- | --- | --- | --- | --- |
| **Sample Name** | **Bioaccessibility (%)** | | | | | | | | | | | |
|  | **Neochlorogenic acid** | | **Chlorogenic acid** | | **Cyanidin-3-*O*-galactoside** | | **Cyanidin-3-*O*-glucoside** | | **Cyanidin-3-*O*-arabinoside** | | **Cyanidin-3-*O*-xyloside** | |
|  | Gastric | Intestine | Gastric | Intestine | Gastric | Intestine | Gastric | Intestine | Gastric | Intestine | Gastric | Intestine |
| AME | 1509.93±12.3 | 428.17±13.4 | 1781.20±2.5 | 532.89±7.5 | 1037.72±2.5 | 30.42±5.6 | 1018.86±6.8 | 28.95±6.4 | 1050.65±43.5 | 42.93±6.4 | 1073.05±23.0 | 40.07±3.6 |
| AME-CP | 2750.83±7.5 | 2777.73±11.4 | 2389.73±21.0 | 2444.30±35.7 | 1185.14±11.2 | 1203.14±6.5 | 1184.57±7.4 | 1238.30±5.8 | 1113.33±9.7 | 1140.92±10.2 | 1189.07±5.7 | 1245.75±7.5 |
| SPI | under LOD | under LOD | under LOD | under LOD | under LOD | under LOD | under LOD | under LOD | under LOD | under LOD | under LOD | under LOD |
| SPI-CP | under LOD | under LOD | under LOD | under LOD | under LOD | under LOD | under LOD | under LOD | under LOD | under LOD | under LOD | under LOD |
| SPI-AME 1:1 | 124.25±5.4 | 34.12±1.9 | 130.19±1.7 | 41.79±6.3 | 85.90±5.7 | 7.45±4.8 | under LOD | under LOD | 68.69±6.3 | 7.54±0.6 | 17.63±3.5 | under LOD |
| SPI-AME CP 1:1 | 106.88±2.4 | 107.82±6.3 | 110.60±7.4 | 111.55±8.9 | 89.41±5.4 | 83.30±8.9 | under LOD | under LOD | 70.14±6.8 | 64.83±5.4 | 20.68±1.3 | 19.18±0.96 |
| SPI-AME 2:1 | 143.17±1.5 | 145.33±5.0 | under LOD | under LOD | 108.08±4.7 | 100.33±6.7 | under LOD | under LOD | under LOD | under LOD | under LOD | under LOD |
| SPI-AME CP 2:1 | 327.32±8.2 | 88.75±3.5 | under LOD | under LOD | 182.07±5.3 | 56.79±0.67 | under LOD | under LOD | under LOD | under LOD | under LOD | under LOD |
| SPI-AME 4:1 | under LOD | under LOD | under LOD | under LOD | 182.0±6.4 | 159.0±5.9 | under LOD | under LOD | under LOD | under LOD | under LOD | under LOD |
| SPI-AME CP 4:1 | under LOD | under LOD | under LOD | under LOD | 233±4.8 | under LOD | under LOD | under LOD | under LOD | under LOD | under LOD | under LOD |
| AME: *Aronia Melanocarpa* Extract, SPI: Soy Protein Isolate, CP: Cold plasma, AME-CP : Cold plasma treated-AME, SPI-CP : Cold plasma treated-SPI, SPI-AME 1:1: SPI-AME interaction at 1:1 ratio *(v/v)*, SPI-AME 2:1: SPI-AME interaction at 2:1 ratio *(v/v)*., SPI-AME 4:1: SPI-AME interaction at 4:1 ratio *(v/v)*, SPI-AME CP 1:1: Cold plasma treated-SPI-AME interaction at 1:1 ratio *(v/v)*, SPI-AME CP 2:1: Cold plasma treated-SPI-AME interaction at 2:1 ratio *(v/v)*, AME CP 4:1: Cold plasma treated-SPI-AME interaction at 4:1 ratio *(v/v)*, Data represent a mean ± SD (n = 3). | | | | | | | | | | | | |
